# Loneliness as a Public Health Challenge: A Systematic Review and Meta-Analysis to Inform Policy and Practice

**DOI:** 10.3390/ejihpe15070131

**Published:** 2025-07-11

**Authors:** Ananda Zeas-Sigüenza, Andreas Voldstad, Pablo Ruisoto, Ana Ganho-Ávila, Raquel Guiomar, Raúl Cacho, Ferran Muntané, Joan Benach

**Affiliations:** 1 Department of Health Sciences, Public University of Navarre, 31006 Pamplona, Spain; ananda.zeas@unavarra.es (A.Z.-S.); raul.cacho@unavarra.es (R.C.); 2 Department of Psychology, School of Psychology and Sport Science, Bangor University, Bangor LL57 2DG, Wales, UK; 3 Department of Psychiatry, Warneford Hospital, University of Oxford, Oxford OX3 7JX, England, UK; andreas.voldstad@kellogg.ox.ac.uk; 4 I-COMMUNITAS, Institute for Advanced Social Research, Public University of Navarre, 31006 Pamplona, Spain; 5 IdisNA, Navarra Institute for Health Research, Public University of Navarre, 31006 Pamplona, Spain; 6 JHU-UPF Public Policy Center (JHU-UPF PPC), UPF Barcelona School of Management (UPF-BSM), Universitat Pompeu Fabra (UPF), 08005 Barcelona, Spain; ferran.muntane@upf.edu; 7 Centre for Research in Neuropsychology and Cognitive Behavioural Intervention, University of Coimbra, 3030-788 Coimbra, Portugal; ganhoavila@fpce.uc.pt (A.G.-Á.); raquelguiomar18@gmail.com (R.G.); 8 Research Group on Health Inequalities, Environment, and Employment Conditions (GREDS-EMCONET), Department of Political and Social Sciences, Universitat Pompeu Fabra, 08005 Barcelona, Spain

**Keywords:** loneliness, meta-analysis, intervention efficacy, baseline severity, public health

## Abstract

Loneliness is a recognized public health risk factor associated with increased morbidity and mortality. However, the effectiveness of interventions targeting loneliness remains unclear—particularly in relation to baseline severity. This systematic review and meta-analysis assessed intervention effectiveness and the influence of baseline severity and intervention characteristics. A total of 25 studies were included, of which 16 randomized controlled trials (RCTs; *k* = 21) were meta-analyzed. Interventions produced a moderate pooled effect at post-intervention (Hedge’s *g* = 0.65, 95% CI [0.05, 1.26], *p* = 0.037), though with high heterogeneity. Sensitivity analyses confirmed a moderate effect (*g* = 0.55, 95% CI [0.22, 0.88], *p* = 0.003). Higher baseline loneliness predicted greater intervention effects (*b* = 0.04, 95% CI [0.02, 0.07], *Z* = 3.36, *p* < 0.001), with cognitive-behavioral therapy (CBT) showing the largest effect size (*g* = 0.73). No significant effects were observed at follow-up. These findings underscore the need for dual strategies: targeted psychological interventions (e.g., CBT) for individuals with severe loneliness, and universal, context-based approaches for the broader population. This aligns with Geoffrey Rose’s distinction between individual-level treatment and population-level prevention and highlights the urgency of embedding loneliness interventions into public health frameworks and policy agendas focused on promoting social connectedness and equity.

## 1. Introduction

Loneliness is a well-established determinant of health, with risks comparable to those of smoking (Holt-Lunstad et al., 2010, 2015) and is increasingly recognized as a critical public health priority (Arsenijevic & Groot, 2018; Holt-Lunstad, 2017; Steptoe et al., 2013). Its prevalence surged following the COVID-19 pandemic, driven by lockdowns and distancing measures (WHO, 2020). As a potent psychosocial stressor, loneliness activates the hypothalamic–pituitary–adrenal axis (J. T. Cacioppo & Hawkley, 2009; S. Cacioppo et al., 2014) and is associated with a wide range of adverse health outcomes, including depression (Vanhalst et al., 2012), suicidal behavior (Mezuk et al., 2014), poor sleep quality (Hom et al., 2020), and cardiovascular disease (Valtorta et al., 2018). Conceptualized as a transdiagnostic factor, loneliness cuts across diagnostic categories and contributes to multiple mental and physical health conditions (Nowland et al., 2018; Ruisoto et al., 2022).

Unlike objective social isolation, loneliness is a subjective experience characterized by a discrepancy between desired and actual meaningful social relationships (J. T. Cacioppo & Patrick, 2008). It exists on a continuum of social connectedness—from low to high or severe loneliness (S. Cacioppo & Cacioppo, 2012)—and is thought to involve altered social information processing, where social interactions may become less rewarding or even aversive in individuals with severe loneliness (Tomova et al., 2020).

Despite its complexity, most intervention studies dichotomize individuals as either lonely or not, often neglecting the degrees in loneliness severity (Astell-Burt et al., 2022; Barreto et al., 2021; Cohen-Mansfield & Perach, 2015; Eccles & Qualter, 2021; Fu et al., 2022; Gardiner et al., 2018; Jarvis et al., 2020; Maes et al., 2019; Masi et al., 2011). Intervention effectiveness is likely influenced by a multitude of factors. These include the intervention focus—whether targeting individual capacities (e.g., enhancing coping or emotion regulation) or the broader context (e.g., addressing environmental or structural conditions) (J. T. Cacioppo & Patrick, 2008; J. T. Cacioppo et al., 2002)—as well as delivery mode (e.g., online vs. in-person), which can impact accessibility, engagement, and the quality of social interaction (Tan et al., 2024; Masi et al., 2011).

Additional moderating factors include intervention format (individual vs. group), which may influence the balance between personalization and social bonding (S. Cohen & Wills, 1985; Hoang et al., 2022), and specific active components such as cognitive restructuring or nature-based activities (Barton & Pretty, 2010; Leavell et al., 2019). Participant characteristics—such as age, gender, and cultural context—can also shape both the experience of loneliness and responsiveness to interventions. For instance, older adults often benefit from interventions that promote meaningful social engagement (de Oliveira et al., 2015), while cultural values (e.g., collectivism vs. individualism) and gender norms can influence how loneliness is conceptualized and mitigated (Hickin et al., 2021; Hoang et al., 2022; Hofstede et al., 2010).

Grounded in this complexity, this study conducts a systematic review and meta-analysis to assess the effectiveness of loneliness interventions and examine how outcomes are moderated by (1) baseline loneliness severity; (2) intervention characteristics (focus, type, delivery mode, format, and active components); and (3) demographic and cultural variables (age, gender, cultural context). This work is informed by Geoffrey Rose’s (1985) influential distinction between strategies that target “high-risk individuals” and those that aim to shift the risk distribution at the population level. By mapping loneliness interventions along this continuum, we aim to provide evidence not only for effective therapeutic approaches, but also for broader public health strategies. Our findings are intended to inform future research, guide mental health service design, and support the development of equity-oriented policies that recognize loneliness as a social determinant of health—shaped by both individual vulnerabilities and structural conditions.

## 2. Materials and Methods

This study was registered in June 2023 (PROSPERO CRD: 42023429666) and followed the guidelines of the Preferred Reporting Items for Systematic Reviews and Meta-Analyses (PRISMA) statement (Page et al., 2021) and the Cochrane Collaboration recommendations (J. P. Higgins et al., 2011; J. P. T. Higgins et al., 2023).

### 2.1. Eligibility Criteria

The primary outcome established for this review was post-intervention loneliness compared to a control group. Studies were eligible if they (1) were published between 2013 and 2023, due to the lack of extensive literature before 2013; (2) were in English or Spanish, the working languages of the review team; (3) employed a controlled quantitative design evaluating the effectiveness of loneliness interventions in adults; and (4) used validated original or short versions of either the gold standard University of Los Angeles Loneliness Scale (UCLA) or the De Jong–Gierveld Loneliness Scale (DJLS). See Supplement 1 for a detailed list of loneliness measures. Only studies involving healthy populations and non-pharmacological interventions were included to minimize confounding variables. Studies using non-standardized loneliness measures or transforming loneliness into binary outcomes were excluded due to limited conceptual comparability.

### 2.2. Search Strategy and Study Selection

Database searches were conducted on 23 June 2023, and repeated on 11 December 2023, in CINAHL, Embase, Medline, PsychINFO, Pubmed, and Web of Science Core Collection (see Supplement 1 for full syntax). Grey literature was identified via ProQuest and OpenAIRE (e.g., theses, dissertations). Backward citation tracking was performed by the first author. Study screening was conducted using Rayyan (Ouzzani et al., 2016). Titles and abstracts were independently screened by two reviewers (A.Z.S & A.V.). Full-text eligibility was independently assessed by two additional reviewers (A.Z.S & R.C.), with disagreements resolved by a third reviewer (P.R.).

### 2.3. Data Extraction and Quality Assessment

Data extraction was piloted and subsequently standardized across all included studies. Extracted information included authors, study design, sample size, baseline characteristics, country and cultural classification following the guidelines of Hofstede et al. (2010), whether loneliness was a primary or secondary outcome, and intervention characteristics (e.g., intervention focus, delivery mode) (see Supplement 1 for full extraction form). Two reviewers (A.Z.S. & R.C.) independently extracted data. Authors from eight studies were contacted for missing information; four provided the requested data and were retained, while the other four were excluded. *Risk of bias* was independently assessed by two reviewers (A.Z.S & R.C.) using the Cochrane Risk of Bias 2.0 tool (RoB) (Sterne et al., 2019), with disagreements resolved by a third reviewer (P.R.).

### 2.4. Statistical Analysis

All analyses were conducted using R (version v4.0.4) using the meta (v4.18.0) and metafor (v2.4.0) packages (Viechtbauer, 2010). Standardized mean differences (SMD) were calculated using Hedges’ *g* with 95% confidence intervals (CIs) for each comparison (*k*), subtracting the mean of the intervention group from the mean of the control group. Positive values indicated less loneliness in the intervention group compared to the control. Magnitude of the difference was classified as small (*g* = 0.20), moderate (*g* = 0.50), or large (*g* ≥ 0.80) (J. Cohen, 1992). A random-effects model was applied, as we assume the effect sizes come from different populations, applying the inverse variance method to calculate the pooled effect size, and standard errors were adjusted with the Hartung–Knapp Adjustment method (IntHout et al., 2016). To address the additional uncertainty of estimating the variance between studies (*τ*^2^), we used the DerSimonian–Laird method to provide the heterogeneity estimate *τ*^2^ (DerSimonian & Kacker, 2007; DerSimonian & Laird, 2015). Hedges’ *g* values were transformed into the number needed to treat (NNT) using the Kraemer and Kupfer (2006) method, where lower NNTs indicate greater effectiveness (Laupacis et al., 1988). Heterogeneity was assessed using Cochran’s *Q* statistic and the proportion of variability due to heterogeneity with the *I*^2^ index, interpreted as low (25%), moderate (50%), or high (75%) (J. P. Higgins et al., 2003). We conducted sensitivity analyses excluding (1) interventions without visual contact (e.g., phone-based or audio-only), due to the role of facial expressions in social information processing; (2) interventions with a combined format (group and individual), to control for the influence of mixed sessions; (3) studies identified with high risk of bias using the Cochrane Risk of Bias (RoB) tool; (4) a study with an effect size significantly larger than the rest in the data set (Cuijpers, 2016); and (5) both the extreme study and the high-RoB studies simultaneously. Publication bias was assessed via visual inspection of funnel plots on loneliness interventions, Begg’s test to assess potential differences between adjusted and observed effect sizes (Begg & Mazumdar, 1994), Egger’s regression test (Egger et al., 1997) of funnel plot asymmetry, and the trim-and-fill procedure (Duval & Tweedie, 2000) to address any potential publication bias. Begg’s and Egger’s tests were rerun post-adjustment to confirm results.

Post hoc subgroup and moderation analyses were conducted for cultural background (Hofstede categories: individualist vs. collectivist societies); intervention focus (person- vs. context-focused); intervention format (individual vs. group); delivery mode (in-person vs. virtual); and loneliness as the primary outcome (yes/no). Interventions were categorized as either single-component (e.g., CBT only) or multi-component interventions (e.g., CBT combined with other techniques).

Meta-regressions were conducted to explore the moderating effects of age, gender, and baseline loneliness severity on the efficacy of loneliness interventions. Moderator significance was assessed using the Q-test for moderators (*QM*). Significant Q-tests of categorical subgroups were interrogated with meta-regressions. For the baseline loneliness severity, all scores were standardized to align with the 20–80 range of the UCLA Loneliness Scale (Version 3). See Supplement 1 (Table S5).

## 3. Results

### 3.1. Characteristics of the Included Studies

**Figure 1:**
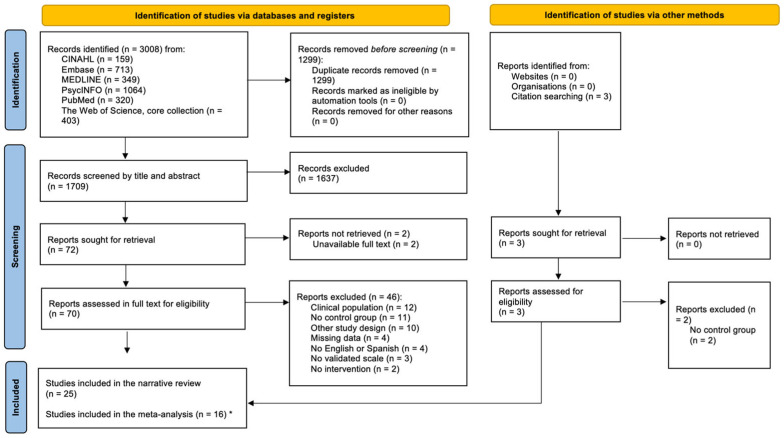
PRISMA flowchart of the study selection process. Of the 25 studies included in the narrative synthesis, 16 were retained for the meta-analysis due to the high heterogeneity of their control groups. * These 16 studies yielded a total of 21 effect sizes and are marked with double asterisks in the references list. “Citation searching” refers to backward reference checking of the included studies.

A total of 25 randomized controlled trials (RCTs) were included in the systematic review. Of these, nine compared loneliness interventions to another intervention (intervention vs. intervention; I-I), and 16 compared loneliness interventions to passive control groups (intervention vs. control; I-C) (see PRISMA flowchart, Figure 1). Across all studies, the total sample at baseline comprised 4761 participants (n Intervention = 2443; n Control = 2318), of whom 71.28% (SD = 15.28%) were women. The mean age was 46.04 years (SD = 22.8) (see Table 1). All studies used the UCLA Loneliness Scale, with one study (Kahlon et al., 2021) additionally using the De Jong–Gierveld Loneliness Scale (DJLS). In 85% of the studies (*n* = 21), loneliness was reported as the primary outcome. Only three studies (12%) applied a pre-settled loneliness severity cut-off as part of their inclusion criteria. Additional information on sample characteristics, intervention description, and follow-up assessments is provided in Table 1 and Supplement 1 (Table S2). Regarding methodological quality, two studies (8%) were assessed as having low risk of bias, nineteen (76%) as moderate risk, and four (16%) as high risk. The primary source of bias (RoB) stemmed from the absence of pre-registered analysis plans. Further detail on risk of bias assessments is available in Supplements 1 and 2.

Narrative results are presented in the following paragraphs. We first present results from intervention–control (I-C) studies (*n* = 16), followed by intervention–intervention (I-I) studies (*n* = 9).

The 16 intervention–control (I-C) randomized controlled trials (RCTs) included a total of 2476 participants at baseline (n Intervention = 1271; n Control = 1205), of whom 68% (SD = 20.8) were women. The mean age across samples was 51.96 years (SD = 21.58). In terms of target populations, 44% (*n* = 7) of studies included older adults, 37.5% (*n* = 6) middle-aged adults, and 12.5% (*n* = 2) undergraduates. Regarding cultural setting, 67% (*n* = 11) of studies were conducted in individualistic cultural settings (Finland, Germany, Israel, Iran, Portugal, Sweden, and the United States) and 33% (*n* = 5) in collectivistic cultural settings (China, Iran, and Turkey). The most common active components were evidence-based psychological interventions, including Cognitive Behavioral Therapy (CBT [31%, *n* = 5]); Interpersonal Psychotherapy (IPT [6%, *n* = 1]); and Acceptance and Commitment Therapy (ACT [6%, *n* = 1]). These were followed by social support interventions (50%, *n* = 8) and reminiscence therapy (12.5%, *n* = 2). In terms of design complexity, 63% (*n* = 10) of studies employed a multiple-component approach: CBT (12.5%, *n* = 2); social support (SS [44%, *n* = 7]); and reminiscence therapy (6%, *n* = 1). Meanwhile, 37.5% (*n* = 6) used a single-component approach, including CBT (19%, *n* = 3), as well as ACT, art therapy, meditation, reminiscence therapy, physical activity, and mindful movement (each 6%, *n* = 1). Most interventions were person focused (81%, *n* = 13), with only 6% (*n* = 1) classified as context–person-focused. Regarding delivery mode, 44% (*n* = 7) were delivered in person, and 56% (*n* = 9) were delivered virtually. Format was evenly split: 50% (*n* = 8) used individual sessions, and 50% (*n* = 8) used group sessions. On average, interventions were delivered over 12.10 sessions (SD = 10.88) within an average period of eight weeks. One study (6%) consisted of a single session, meanwhile two studies (12.5%) spanned one month and six months, respectively.

The nine intervention–intervention (I-I) randomized controlled trials (RCTs) included a total of 2258 participants at baseline (n Intervention = 1158; n Control = 1100), of whom 73% (SD = 7.66) were women. The mean age across samples was 34.80 years (SD = 20.44). In terms of sample composition, 4 studies focused on undergraduate students, 3 on older adults, and 1 on middle-aged adults. Regarding cultural context, 6 studies were conducted in individualistic cultural settings (United Kingdom & United States), and 3 in collectivistic cultural settings (Taiwan). The most common active component was interventions that promoted social interactions (*n* = 2), followed by mindfulness-based interventions (*n* = 2). Other components—each represented in a single study—included art-therapy, volunteering, nature exposure, virtual interaction with strangers, and digital social support. Most interventions (*n* = 7) used a single-component approach, while 2 studies used a multiple-component approach. Only one study examined the effect of a context-based intervention. Delivery modes were predominantly virtual (*n* = 7); and two were delivered in-person. Most were individual-based (*n* = 8), with only one delivered in a group format. On average, I-I interventions were delivered in 12.10 sessions (SD = 10.88), over a span of approximately 13 weeks. Two studies represented temporal outliers, with interventions delivered within four days or extended across 12 months, respectively. Regarding outcomes, six of the I-I RCTs reported statistically significant pre-post reductions in loneliness, two reported no significant changes, and one did not report *p*-values. For detailed *p*-values, see Supplement 1.

### 3.2. Meta-Analysis

**Figure 2:**
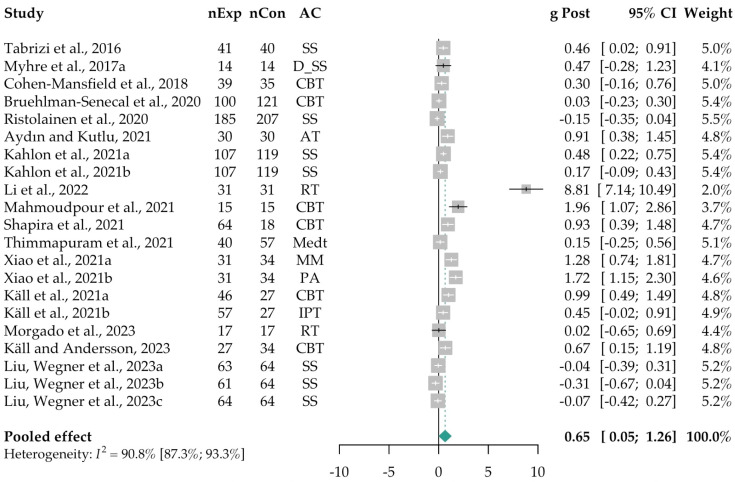
Forest plot of the effect sizes of interventions vs. control groups (16 Intervention–Control [I-C] RCTs; *k* = 21) at post intervention. A random-effects model with Hartung–Knapp adjustment was used to provide more accurate standard errors. Tabrizi et al. (2016) (*k* = 1); Myhre et al. (2017) ^a^ (*k* = 1); Cohen-Mansfield et al. (2018) (*k* = 1); Bruehlman-Senecal et al. (2020) (*k* = 1); Ristolainen et al. (2020) (*k* = 1); Aydın and Kutlu (2021) (*k* = 1); Kahlon et al. (2021) (*k* = 2; Kahlon et al., 2021 ^a,b^); Li et al. (2022) (*k* = 1); Mahmoudpour et al. (2021) (*k* = 1); Shapira et al. (2021) (*k* = 1); Thimmapuram et al. (2021); Xiao et al. (2021) (*k* = 2; Xiao et al., 2021 ^a,b^); Käll et al. (2021) (*k* = 2; Käll et al., 2021 ^a,b^); Morgado et al. (2023) (*k* = 1); Käll and Andersson (2023) (*k* = 1); S. Liu et al. (2023) (*k* = 3; S. Liu et al., 2023 ^a–c^). Abbreviations: AC = Main Active Component used in each intervention; AT = Art Therapy; CBT = Cognitive Behavioral Therapy; CI = Confidence Interval; D_SS = Digital Social Support; *g* Post = Hedges’ *g* at post-intervention; *I*^2^ = Heterogeneity; IPT = Internet-based Interpersonal Therapy; Medt = Meditation; MM = Mindful Movement; nExp = Experimental group sample size; nCon = Control group sample size; PA = Physical Activity; RT = Reminiscence Therapy; SS = Social Support; *k* = number of comparisons per intervention; ^a^ = first comparison, ^b^ = second comparison, ^c^ = third comparison.

Due to the substantial heterogeneity in main active components across the intervention-intervention (I-I) RCTs, it was not feasible to group or compare them with consistent control types. As a result, only the 16 intervention-control (I-C) RCTs were included in the meta-analysis. The overall pooled effect size of loneliness interventions was moderate, with Hedges’ *g* = 0.65 (95% CI [0.05; 1.26]), *p* = 0.037), and a number needed to treat (NNT) of 2.82. However, heterogeneity was high, with *Q*_(df=20)_ = 216.75, *p* < 0.001, and *I*^2^ = 91% (95% CI [87; 93]) (see Figure 2). One study (Li et al., 2022) exhibited an extremely large effect size (*g* = 8.81, 95% CI [7.14; 10.49]), prompting sensitivity analyses that excluded this study. Follow-up data were available in six studies (*k* = 8 comparisons). The pooled follow-up effect size was non-significant, *g* = 1.91 (95% CI [−1.87; 5.69], *p* = 0.251), with very high heterogeneity (*Q*_(df=5)_ = 113.27, *p* < 0.001; *I*^2^ = 95.6%, 95% CI [93; 97]). For detailed results of the follow-up effects, see Supplement 1 (Table S3) and Supplement 3.

Three sensitivity analyses were conducted to assess the robustness of post-intervention effects. These included removing a study with a mixed intervention format, excluding two studies due to the nature of the online intervention, and excluding studies with high risk of bias (RoB) (Morgado et al., 2023; Myhre et al., 2017; Ristolainen et al., 2020; Thimmapuram et al., 2021). In all cases, the pooled effect sizes at post-intervention remained moderate. After excluding the study with an extremely large effect size (*g* = 8.81), the pooled effect was small to moderate, *g* = 0.46 (95% CI [0.19; 0.73], *p* = 0.002, NNT = 3.92), with high heterogeneity, *Q*_(df=19)_ = 116.73, *p* < 0.001; *I*^2^ = 84% (95% CI [76; 89]). When both the extreme effect size study and the high RoB studies were excluded, the pooled effect size remained moderate, *g* = 0.55 (95% CI [0.22; 0.88], *p* = 0.003, NNT = 3.29), with similarly high heterogeneity, *Q*_(df= 15)_ = 95.41, *p* < 0.001; *I*^2^ = 84% (95% CI [76; 89]). Follow-up effects remained non-significant across all sensitivity analyses. For full sensitivity analysis results, see Supplement 1 (Table S3) and Supplement 4.

### 3.3. Risk of Publication Bias

Publication bias was assessed using both the full sample of studies and a subset of 11 RCTs (after excluding the study with an extreme effect size and those rated as high risk of bias). In the full sample, both Begg’s rank correlation test and Egger’s regression test were statistically significant, indicating potential publication bias (see Supplement 1 for full details). The trim and fill method estimated that seven studies were potentially missing, resulting in an adjusted total of 23 studies. After adjustment, the corrected random-effects model showed a non-significant effect size, *g* = 0.14 (95% [−0.62, 0.91], *p* = 0.698), with high heterogeneity, *Q*_(df=17)_ = 431.03, *p* < 0.001, *I*^2^ = 93.7%. After correction, Begg’s and Egger’s tests were no longer significant, further supporting the bias adjustment. In the subset analysis (excluding extreme- and high-RoB studies), Begg’s and Egger’s tests were again initially significant, suggesting small-study effects (see Supplements 1 and 5). The trim and fill method identified six potentially missing studies, increasing the total to 22 studies. The corrected effect size was again non-significant and small, *g* = 0.19 (95% CI [−0.20; 0.58], *p* = 0.328), with high heterogeneity, *Q*_(df=21)_ = 194.1, *p* < 0.001; *I*^2^ = 89%. After this adjustment, both tests for publication bias became non-significant. In summary, these findings suggest that smaller studies with larger effect sizes may be overrepresented, and caution is warranted in interpreting pooled results. See Supplements 1 and 5 for additional details.

### 3.4. Moderation and Subgroup Analyses

**Figure 3:**
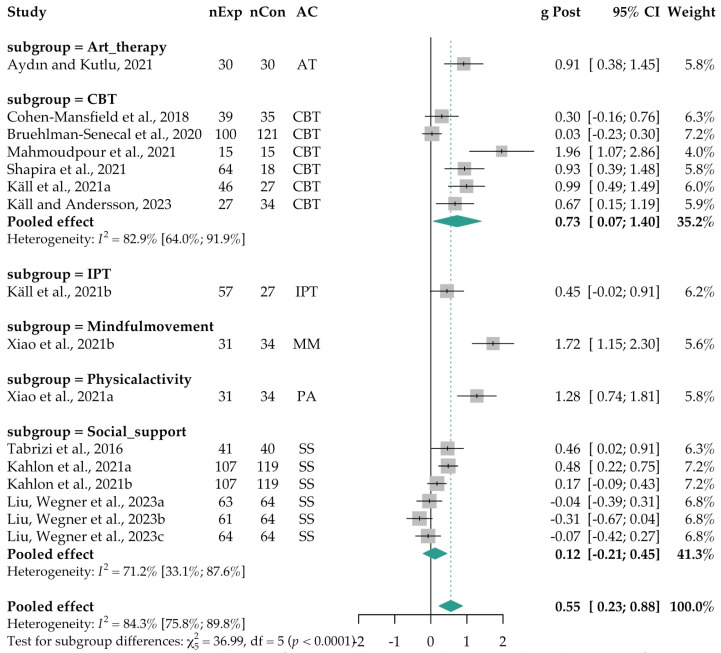
Forest plot of the effect sizes of the interventions vs. controls post-intervention: Subgroup analysis by active component (*k* = 16). A random-effects model with Hartung–Knapp adjustment was applied to provide more accurate standard error estimates. Tabrizi et al. (2016) (*k* = 1); Cohen-Mansfield et al. (2018) (*k* = 1); Bruehlman-Senecal et al. (2020) (*k* = 1); Aydın and Kutlu (2021) (*k* = 1); Kahlon et al. (2021) (*k* = 2; Kahlon et al., 2021 ^a,b^); Li et al. (2022) (*k* = 1); Mahmoudpour et al. (2021) (*k* = 1); Shapira et al. (2021) (*k* = 1); Xiao et al. (2021) (*k* = 2; Xiao et al., 2021 ^a,b^); Käll et al. (2021) (*k* = 2; Käll et al., 2021 ^a,b^); Käll and Andersson (2023) (*k* = 1); S. Liu et al. (2023) (*k* = 3; S. Liu et al., 2023 ^a–c^). Abbreviations: AC = Main Active Component used in each intervention; AT = Art Therapy; CBT = Cognitive Behavioral Therapy; CI = Confidence Interval; df = Degrees of Freedom; *g* Post = Hedges’ *g* at post-intervention; *I*^2^ = Heterogeneity; IPT = Internet-based Interpersonal Therapy; MM = Mindful Movement; nExp = Experimental group sample size; nCon = Control group sample size; PA = Physical Activity; SS = Social Support; *k* = number of comparisons per intervention; ^a^ = first comparison, ^b^ = second comparison, ^c^ = third comparison.

Subgroup analyses and meta-regressions for age and gender were conducted using the subset of 11 studies (*k* = 16 comparisons) to strengthen the reliability of results. A significant difference was observed for cultural orientation, based on Hofstede’s classification (*Q* = 9.92, *p* < 0.001). In the subsequent subgroup analysis, interventions were found to be more effective in collectivistic cultures compared to individualistic ones (*k* = 5; *b* = 0.88, 95% CI [0.32, 1.45], *p* = 0.004). The pooled effect size was large and significant for collectivism-based studies (*k* = 5, *g* = 1.21, 95% CI [0.47; 1.95]), and small but significant for individualism-based studies (*k* = 11; *g* = 0.29, 95% CI [0.01; 0.56]). Significant differences were also found based on whether loneliness was assessed as a primary versus secondary outcome (*Q* = 16.80, *p* < 0.001). In subgroup analysis, interventions were more effective when loneliness was a secondary outcome rather than the primary focus (*k* = 14; *b* = −1.08, 95% CI [−1.96, −0.21, *p* = 0.018]). The pooled effect size was moderate and significant for studies measuring loneliness as a secondary outcome (*k* = 14; *g* = 0.41, 95% CI [0.11; 0.71]) but not significant for those with loneliness as the primary outcome (*k* = 2; *g* = 1.49, 95% CI [−1.35; 4.33]). Finally, a significant difference was found based on the main active component used in interventions (*Q* = 36.99, *p* < 0.001) (see Figure 3).

We interrogated the differences between the largest active component subgroups—CBT-based interventions (including ACT) and social support interventions. The meta-regression showed a trend favoring CBT, though it did not reach statistical significance (*k* = 7; *b* = 0.57, 95% [−0.04, 1.18], *p* = 0.062). A significant pooled effect was found for the CBT subgroup (*k* = 7; *g* = 0.73, 95% CI [0.07; 1.40]) but not for the social support subgroup (*k* = 6; *g* = 0.12, 95% CI [−0.21; 0.45]). Among smaller subgroups, significant effects were observed for art therapy (*k* = 1; *g* = 0.91, 95% CI [0.38; 1.45]), mindful movement (*k* = 1; *g* = 1.72, 95% CI [1.15; 2.30]), and physical activity (*k* = 1; *g* = 1.28, 95% CI [0.74; 1.81]). However, these results should be interpreted with caution, as they are derived from single-study estimates rather than pooled subgroup data. For the full set of subgroup analyses, see Supplement 1 (Table S4) and Supplement 6. No significant differences were found in subgroup analyses by intervention focus (person-focused vs. context-focused) (*Q* = 0.89, *p* = 0.346), delivery mode (in-person vs. virtual) (*Q* = 0.73, *p* = 0.391), or intervention format (group vs. individual) (*Q* = 1.39, *p* = 0.239) (see Supplements 1 and 6 for full results).

**Figure 4:**
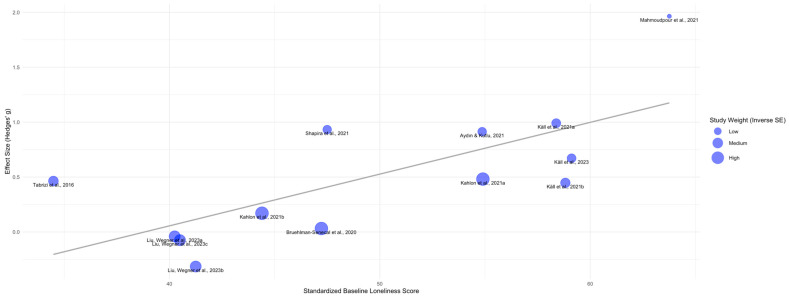
Association between baseline loneliness severity and intervention efficacy (*k* = 16). The bubble plot depicts the association between standardized baseline loneliness scores (x-axis) and intervention effect sizes (Hedges’ *g*) (y-axis). Each blue bubble corresponds to a study. Bubble size reflects the inverse of the study’s standard error, meaning larger bubbles indicate studies with greater precision. The gray trend depicts a positive association, revealing that higher levels of baseline loneliness are linked to larger intervention effects. The legend, “Study Weight (Inverse SE)”, categorizes study precision from “Low” to “High”. Tabrizi et al. (2016) (*k* = 1); Bruehlman-Senecal et al. (2020) (*k* = 1); Aydın and Kutlu (2021) (*k* = 1); Kahlon et al. (2021) (*k* = 2; Kahlon et al., 2021 ^a,b^); Shapira et al. (2021) (*k* = 1); Mahmoudpour et al. (2021) (*k* = 1); Käll et al. (2021) (*k* = 2; Käll et al., 2021 ^a,b^); Käll and Andersson (2023) (*k* = 1); S. Liu et al. (2023) (*k* = 3; S. Liu et al., 2023 ^a–c^); *k* = number of comparisons per intervention; ^a^ = first comparison, ^b^ = second comparison, ^c^ = third comparison.

A significant negative association was found between the proportion of women participants and intervention effect size (*b* = −0.01, SE = 0.01, 95% CI [−0.03, 0.01]; *t*_(df=14)_ = −1.11, *p* = 0.026). This indicates that a 1% increase in the proportion of women in a study sample was associated with a −0.01 reduction in the intervention effect size. However, the model explained a very small proportion of variance (*R*^2^ = 0.50%) and exhibited high residual heterogeneity (*I*^2^ = 84.37%, *τ*^2^ = 0.22, SE = 0.11) (see Supplement 7). Age was not a significant moderator (*b* = 0.00, SE = 0.01, 95% CI [−0.02, 0.01], *t*_(df=14)_ = −0.34, *p* = 0.735). Two studies were excluded from the final meta-regression due to inconsistencies in their reported loneliness scores: Cohen-Mansfield et al. (2018) averaged across three different scales without providing UCLA loneliness raw scores, and Xiao et al. (2021) showed discrepancies between reported loneliness scores and the Likert scale used. The final model revealed a significant positive association between baseline loneliness scores and intervention efficacy (*b* = 0.04, SE = 0.01, 95% CI [0.02, 0.07]; *Z* = 3.36, *p* = < 0.001). Each one-unit increase in standardized baseline loneliness scores was associated with a 0.04-point increase in effect size. This model accounted for a substantial proportion of between-study variance (*R*^2^ = 59.04%), though residual heterogeneity remained high (*QE*_(df=11)_ = 32.09, *p* = < 0.001; *τ*^2^ = 0.09 (SE = 0.06), (*I*^2^ = 70.19%) (see Figure 4 and Supplement 1 Table S5).

## 4. Discussion

This review yields three major findings with important implications for research, practice, and public health policy. First, it confirms the overall effectiveness of loneliness interventions in reducing subjective experiences of loneliness. Second, intervention outcomes vary substantially depending on the specific active components employed. Third—and most notably—interventions are significantly more effective for individuals experiencing higher baseline levels of loneliness. To our knowledge, this is the first meta-analysis to systematically examine this moderating effect across a diverse set of trials.

A key finding is the substantial heterogeneity in both intervention types and outcomes. Many interventions combined multiple active components, especially those focused on enhancing interpersonal relationships through social support. This complexity hinders the identification of potential causal mechanisms, as well as the isolation of intervention-specific effects. Moreover, considerable variation in intervention duration and intensity further challenges interpretation. While shorter interventions may benefit from novelty or greater accessibility, longer ones might gain effectiveness from providing sustained opportunities for meaningful connection—regardless of therapeutic content.

The absence of significant differences in outcomes based on delivery mode (in-person vs. virtual) and format (group vs. individual) warrants consideration. One possible explanation is the limited power of these analyses due to high heterogeneity and small sample sizes. Another potential explanation is that virtual and group-based interventions have improved in quality, accessibility, and engagement, particularly in the post-COVID-19 era. A further consideration is that some of the studies were conducted during the COVID-19 pandemic. In the context of limited social interactions, virtual interactions might have served as a temporary replacement for face-to-face interactions during the COVID-19 lockdowns. The scarcity of social interactions and the active component of the intervention may outweigh any effect of the delivery mode. The comparisons of virtual and in-person delivery modes, and group and individual intervention formats need more rigorous and controlled studies. Three-arm RCTs could be a viable design to compare the interventions with active and passive controls simultaneously, to address the potential influence of a placebo effect, and to determine which interventions are most effective for different individuals.

Among person-focused strategies, Cognitive Behavioral Therapy (CBT) showed the strongest and most consistent evidence of effectiveness, aligning with previous reviews (Masi et al., 2011; Hickin et al., 2021). Other individual-level approaches, such as art therapy and movement-based interventions, also showed promise—though evidence remains limited to single studies. Surprisingly, social support interventions—often regarded as central to loneliness mitigation—did not show a significant pooled effect. This may reflect their episodic nature, or the difficulty severely lonely individuals face in engaging in or benefiting from interpersonal interactions (Tomova et al., 2020; Gardiner et al., 2018).

In contrast, reminiscence therapies were constrained by moderate risk of bias and inflated effect sizes. One study (Li et al., 2022) reported an exceptionally large effect size and moderate risk of bias. This intervention was highly intensive and culturally tailored, combining reminiscence-based activities with traditional practices for older adults in rural China. Notably, participants were familiar with one another prior to the intervention, which may have enhanced its impact by fostering stronger group cohesion and emotional resonance. While we included this study in the main analysis for completeness, we conducted two of the five sensitivity analyses excluding it. The overall results remained robust, indicating that the main findings are not overly influenced by the study.

While contextual and nature-based interventions were underrepresented in the current evidence base, they offer considerable potential as scalable, population-level strategies. Rooted in the biophilia hypothesis (Wilson, 1986), these interventions leverage the innate human affinity for natural environments to promote psychological well-being and social connection. Emerging evidence suggests they may reduce loneliness by up to 28% (Hammoud et al., 2021). Their relevance is heightened in the context of the current ecosocial crisis, where climate change, biodiversity loss, and urban stressors intersect with growing psychosocial vulnerability. These strategies offer co-benefits for individual health, environmental restoration, and social cohesion (Whitmee et al., 2015; Lang & Rayner, 2012).

Such approaches are increasingly recognized in public health policy. The UK’s Campaign to End Loneliness and the NHS’s Social Prescribing initiative explicitly link community-based and green interventions to mental health and social integration (NHS England, 2019). Similarly, the WHO Urban Health Initiative promotes urban design that fosters social cohesion and well-being (WHO, 2016). These policy directions align with Geoffrey Rose’s (1985) classic distinction between high-risk and population-level strategies of prevention. From this perspective, CBT functions as a targeted secondary intervention for individuals experiencing severe loneliness, while universal and context-based interventions function as primary prevention, shifting the population distribution of loneliness. Importantly, integrating both levels is essential not only for clinical and economic impact, but also for ethical and public health reasons—addressing both individual suffering and structural determinants.

Our findings support this integrative model. Meta-regression results confirmed baseline loneliness severity as a strong moderator of intervention effectiveness. Although regression to the mean may partially account for this association, the analysis was restricted to a subset of studies with lower risk of bias and excluded studies with inconsistencies in their reported loneliness scores and extreme values. Tailoring intensity and delivery to individual needs is thus clinically, economically, and ethically justified. The presence of floor and ceiling effects also underscores the need to match interventions to baseline severity—both to prevent resource misallocation and to optimize measurable outcomes.

Measurement issues also warrant attention. The UCLA Loneliness Scale appeared more responsive to intervention effects than alternative tools (e.g., DJLS), consistent with previous reviews (Masi et al., 2011). A refined classification—for example, defining “severely lonely” as UCLA scores ≥65—could enhance targeting and outcome measurement by extending Perry’s (1990) classification. This recommendations are based on the loneliness baseline scores of the included studies, the scarcity of pre-settled loneliness inclusion criterion cut-offs, and the increasing rates of loneliness especially since 2020. Moreover, the frequent co-occurrence of depressive symptoms suggests a need for integrated assessment and intervention approaches.

Sociodemographic moderators were also relevant. Interventions were more effective in collectivistic cultural settings, suggesting that societal norms around interdependence may amplify intervention salience (Lykes & Kemmelmeier, 2014). One potential explanation is that collectivist cultures emphasize interdependence, group cohesion, and mutual support, which may enhance engagement with interventions that promote social connection by shaping how people respond emotionally and attitudinally to these experiences (Taras et al., 2010). Moreover, a recent meta-analysis (Wang et al., 2024) evidenced that these cultural dimensions can moderate the associations between loneliness and psychosocial outcomes, including social anxiety and stress. These findings suggest that cultural context not only shapes baseline experiences of loneliness but may also influence how individuals respond to interventions designed to alleviate it.

A modest gender effect was observed, with interventions yielding greater benefits in studies with a higher proportion of men, echoing earlier findings (Hickin et al., 2021; Masi et al., 2011). These findings highlight the importance of gender-sensitive and culturally informed intervention strategies, and future research should also consider socioeconomic position and material conditions, which are central to the social determinants of loneliness.

The higher average age in the I–C group may partly explain their greater responsiveness to certain interventions. As individuals age, their social goals shift toward emotionally meaningful interactions, a pattern described by Socioemotional Selectivity Theory (Carstensen et al., 2003). According to this framework, older adults tend to prioritize close, emotionally satisfying relationships over broader social networks. This theoretical framework may explain both the nature of loneliness in older populations and the effectiveness of interventions that emphasize emotional connection, suggesting that age-related differences in social priorities could enhance intervention outcomes.

Finally, several limitations must be acknowledged. First, the marked heterogeneity of interventions limits our ability to isolate specific mechanisms of change. Second, some studies presented dependent effect sizes (e.g., across time points), which may bias pooled estimates but could not be fully modelled due to dataset size. Third, the scarcity of follow-up data precludes firm conclusions about the long-term sustainability of intervention effects. Fourth, although the search strategy was broad, studies using newer loneliness measures or published after the review window may have been missed. Fifth, methodological quality varied across studies, with some exhibiting scoring discrepancies, extreme effect sizes, or inadequate reporting of randomization and analysis plans. For future reviews, the combined use of INSPECT-SR (Wilkinson et al., 2023) and the Cochrane Risk of Bias Tool is recommended to enhance trustworthiness and transparency.

## 5. Conclusions

This study offers four critical insights into the effectiveness and design of loneliness interventions. First, interventions are significantly more effective among individuals with higher levels of loneliness, underscoring the importance of stratified approaches that tailor intensity and content to severity levels. Second, CBT-based interventions emerge as robust secondary prevention strategies, particularly effective for individuals experiencing severe loneliness. Third, nature-based interventions show promise as universal primary prevention strategies, especially for mitigating mild loneliness and enhancing social connectedness; however, further evidence from well-controlled trials is needed. Fourth, outcomes appear to vary by gender, with preliminary evidence suggesting greater benefit among men, highlighting the need for culturally and demographically responsive intervention designs. Taken together, these findings support a dual-strategy public health model aligned with Rose’s prevention framework. Future research should integrate methodological rigor, equity considerations, and ecological awareness to enhance both the scientific validity and the societal relevance of loneliness interventions.

## Figures and Tables

**Table 1:** Characteristics of the 25 reviewed studies.

Study RoB: Overall Risk	Sample (Type of Sample) % Women Mean Age (SD)	Country (Culture)	Contrast: *Main Active Component* vs. CG Type	Duration: # Sessions (Length per Session	Delivery-Mode; Format; Focus (Centered)	Follow-Up (Months)	Severity-Based Inclusion Criteria
**Aydın and Kutlu (2021)** RoB: Some concerns	60 (Older Adults) 78.3% 72.56 (±1.01)	Turkey (Coll)	*Art therapy* vs. NI	×6 (60–90 min); length (n/r)	In person; group; person-centered	No	Yes
**Bruehlman-Senecal et al. (2020)** RoB: Some concerns	221 (College Students) 59.3% 18.68 (0.35)	United States (Ind)	*CBT* vs. DA	n/r (n/r); 4 weeks	Virtual; individual; person-centered	No	Yes
**Cohen-Mansfield et al. (2018)** RoB: Some concerns	74 (Older Adults) CBT 79.49% NI 82.86% CBT 76.6 (6.8) NI 79 (6.62)	Israel (Coll)	*CBT* vs. NI	×7 individual (n/r) ×10 group (n/r); length (n/r)	In person; group; person-centered	Yes (3)	No
**Kahlon et al. (2021)** RoB: Low	240 (Older Adults) 79% SS 69.4 (11.5) NI 68.7 (12.8)	United States (Ind)	*Social Support* vs. NI	×20 (~less than 10 min; 5 per week); 4 weeks	Virtual; individual; person-centered	No	No
**Käll and Andersson (2023) ^c^** RoB: Some concerns	73 (Adults) 71.2% 47.2 (17.63)	Sweden (Ind)	*CBT* vs. DA	×8 (n/r); 8 weeks	Virtual; individual; person-centered	Yes (24)	Yes
**Käll et al. (2021)** ^b^ RoB: Some concerns	170 (Adults) 75.9% 47.5 (16.4)	Sweden (Ind)	*CBT* vs. *WL* *IPT* vs. *WL*	×9 (n/r); 9 weeks	Virtual; individual; person-centered	Yes (4)	No
**Li et al. (2022)** RoB: Some concerns	64 (Older Adults) 63.3% 65.7 (3.69)	China (Coll)	*Reminiscence therapy* vs. NI	×8 (4 h); length (n/r)	In person; group; person-centered	Yes (3)	No
**S. Liu et al. (2023)** RoB: Some concerns	252 (Adults) 73% 33.93 (11.84)	Germany (Ind)	*Social support* vs. NI	×1 (~4 min); 1 day	Virtual; individual; person–context-centered	No	No
**Mahmoudpour et al. (2021)** RoB: Some concerns	83 (Divorced Women) 100% 32 (n/r)	Iran (Coll)	*ACT* vs. NI	×8 (90 min); 1 month	In person; group; person-centered	No	No
**Morgado et al. (2023)** RoB: High	34 (Older Adults) 50% 81.97 (8.03)	Portugal (Ind)	*Reminiscence therapy* vs. NI	×10 (45 min); length (n/r)	In person; group; person-centered	No	No
**Myhre et al. (2017)** ^a^ RoB: High	121 (Older Adults) D-SS 36% OD 31% WL 21% D-SS 80 (7.34) OD 78.38 (7.32) WL 79.29 (6.76)	United States (Ind)	*Digital-Social Support* vs. WL	nr (n/r); 8 weeks	Virtual; individual; person-centered	No	No
**Ristolainen et al. (2020)** RoB: High	392 (Older Adults) 82.9% 76.8 (7.5)	Finland (Ind)	*Social support* vs. NI	×5 (2–3 h); 6 months	In person; group; person-centered	No	No
**Shapira et al. (2021)** RoB: Some concerns	82 (Older Adults) CBT 81% WL 78% CBT 72.1 (5.3) WL 71.7 (6.8)	Israel (Ind)	*CBT* vs. WL	×7 (60–90 min); ~4 weeks	In person; group; person-centered	No	No
**Tabrizi et al. (2016)** RoB: Some concerns	140 (Breast Cancer survivors) 100% 47.9 (11.4)	Iran (Coll)	*Social Support* vs. NI	×12 (90 min); 12 weeks	In person; group; person-centered	Yes (2)	No
**Thimmapuram et al. (2021)** RoB: High	155 (Physicians) 66% 46 (11.03)	United States (Ind)	*Meditation* vs. DA.	×~28 Sessions (15 min); 4 weeks	Virtual; individual; person-centered	No	No
**Xiao et al. (2021)** RoB: Some concerns	96 (University Students) ~26.04% PA 18.95 (0.89) MM 19.21 (1.02) NI 19.71 (1.77)	China (Coll)	*Physical Activity* vs. NI *Mindful Movement* vs. NI	×36 (90 min); 12 weeks	Virtual; individual; person-centered	No	No
Hussain et al. (2023) RoB: Some concerns	110 (College students) 79% 20.97(4.83)	United Kingdom (Ind)	*Mindfulness* vs. Happier Life Intervention	nr (n/r); 4 weeks	Virtual; individual; person-centered	Yes (0.5)	No
Lanser and Eisenberger (2023) ** RoB: Some concerns	300 (Undergraduate students) 79.7% 20.08 (n/r)	United States (Ind)	*Prosocial behavior-Giving* vs. Logo viewing *Prosocial behaviour-Keeping* vs. Logo viewing	×1 (task done only once); length (n/r)	Virtual; individual; person-centered	No	No
Lanser and Eisenberger (2023) ** RoB: Some concerns	300 (Undergraduate students) 77.1% 20.50 (n/r)	United States (Ind)	*Prosocial behavior-Gratitude* vs. TV-show writing *Prosocial behaviour-Reflection* vs. TV-show writing	×1 (task done only once); length (n/r)	Virtual; individual; person-centered	No	No
Lindsay et al. (2019) RoB: Some concerns	153 (Young Adults) 67.32% 32.42 (13.68)	United States (Ind)	*Mindfulness* vs. Coping control	×14 sessions (20 min); 2 weeks	Virtual; individual; person-centered	No	No
C. W. Liu et al. (2023) RoB: Some concerns	100 (Older Adults) D-SS 74% YT-i 70% D-SS 72.90 (4.45) YT-i 72.78 (±4.52)	Taiwan (Coll)	*Digital-Social Support* vs. YouTube Interaction	×60 (1 h); 12-weeks	Virtual; group; person-centered	No	No
Razani et al. (2018) RoB: Some concerns	78 (Child-parent pairs) 87% 38 (n/r)	United States (Ind)	*Nature exposure* vs. Independent Park prescription	×3 (n/r); 3 weeks	In person; individual; context-centered	No	No
Van Orden et al. (2022) RoB: Low	291 (Older Adults) 75.3% 72 (9.07)	United States (Ind)	*Volunteering* vs. Life Review	n/r (n/r); 12 months	In person; individual; person-centered	No	No
Yang et al. (2023) RoB: Some concerns	90 (Older Adults) 64.04% VI 68.07 (6.68) N-On 69.00 (6.04)	Taiwan (Coll)	*Virtual interaction with strangers* vs. Non-online interaction group	×40 (n/r); 8 weeks	Virtual; individual; person-centered	No	No
Zhang et al. (2023) RoB: Some concerns	132 (University Students) 62.9% 24.4 (10.1)	United States (Ind)	*Art therapy* vs. Hobbies-writing	×4 (15 min); 4 days	Virtual; individual; person-centered	No	No

Notes: Bolded study ID indicates Intervention–control (I-C) design-based studies selected in the meta-analysis (n = 16). ^a^ Only one comparison from this study was included in the meta-analysis. ^b^ Excluded from follow-up analyses as follow-up data was collected only for the experimental group. ^c^ Data from this intervention was also retrieved from (Käll et al., 2020a, 2020b). ** Indicates multiple studies reported in the same publication. Abbreviations: ACT = Acceptance and Commitment Therapy; CBT = Cognitive Behavioral Therapy; CG = Control Group; Coll = Collectivistic; DA = Delayed Access; D-SS = Digital Social Support; Ind = Individualistic; IPT = Internet-based Interpersonal Therapy; MM = Mindful Movement; NI = No Intervention; N-On = Non-online Interaction Group; n/r = number of sessions not reported, and/or length, and/or age not reported; OD = Online Diary; PA = Physical Activity; RoB = Risk of Bias assessment; SS = Social Support; VI = Virtual Interaction with Strangers; WL = Waiting List; YT-i = YouTube interaction; # = number of sessions in each intervention.

## Data Availability

The raw data extracted from the included studies and R code are openly available in the Open Science Framework (OFS) and can be accessed at https://osf.io/d3rak/?view_only=3dd83d8426884b6885f547e78953e853 (accessed on 30 June 2025).

## Supplementary Materials

The following supporting information can be downloaded at: https://www.mdpi.com/article/10.3390/ejihpe15070131/s1, Supplement 1: syntax, inclusion criteria, data extraction form, risk of publication bias details, tables, loneliness’ scales references; Supplement 2: Risk of Bias overall results, Figures S1 and S2; Supplement 3: Follow-up forest plot, Figure S3; Supplement 4: Forest plots of the five sensitivity analyses, Figures S4–S8; Supplement 5: Funnel plots, Figures S9 and S10; Supplement 6: Forest plots of the subgroup analyses, Figures S11–S15; Supplement 7: Association between gender and intervention efficacy, Figure S16.

## Abbreviations

The following abbreviations are used in this manuscript:

AC	Main Active Component used in the intervention
ACT	Acceptance and Commitment Therapy
AT	Art Therapy
CBT	Cognitive Behavioral Therapy
CI	Confidence Interval
CG	Control Group
Coll	Collectivistic
DA	Delayed Access
DJLS	de Jong–Gierveld Loneliness scale
D-SS	Digital Social Support
I-C	Intervention vs. Control
I-I	Intervention vs. Intervention
Ind	Individualistic
INSPECT-SR	INveStigating ProblEmatic Clinical Trials in Systematic Reviews
IPT	Internet-Based Interpersonal Therapy
MM	Mindful Movement
Medt	Meditation
NHS	National Health System
NI	No Intervention
NNT	Number Needed to Treat
N-On	Non-online Interaction Group
N/r	Number of sessions not reported
n/r	Length or age not reported
OD	Online Diary
OSF	Open Science Framework
PA	Physical Activity
PRISMA	Preferred Reporting Items for Systematic Reviews and Meta-Analyses
RCT	Randomized Controlled Trial
RoB	Risk of Bias
SS	Social Support
SMD	Standardized Mean Differences
UCLA (v3)	University of Los Angeles Loneliness scale (version three)
UK	United Kingdom
VI	Virtual Interaction with Strangers
WL	Waiting List
WHO	World Health Organization
YT-i	YouTube interaction
