# Supplementary material for: Loneliness as a Public Health Challenge: A Systematic Review and Meta-Analysis to Inform Policy and Practice
Source: Eur J Investig Health Psychol Educ. 2025 Jul 11;15(7):131. doi: 10.3390/ejihpe15070131 (PMC12293955; doi:10.3390/ejihpe15070131)
Supplement: Supplementary file 1 [file ejihpe-15-00131-s001.zip › Supplement 1_tables_loneliness scale references.pdf]

Supplementary material

Raw data and R code:

To access raw data extracted from the included studies and R code please go the following link: https://osf.io/d3rak/?view_only=3dd83d8426884b6885f547e78953e853

Search Strategy Syntax

Databases search used the following search syntax: ("social isolation" OR lonel* OR "lonely people") AND (reduc* OR minimis* OR minimiz* OR ameliorat* OR intervention* OR program* OR treatment* OR therap* OR "green areas" OR "urban areas" OR medit* OR psychotherapy* OR mindful*) AND ("ucla scale" OR "UCLA loneliness scale" OR " Gierveld scale").

List of Loneliness Measures for Inclusion Criteria

University of California Los Angeles, Loneliness scale (UCLA; 20 items) (Russell et al., 1978); Revised University of California Los Angeles, Loneliness scale (R-UCLA; 20 items) (Russell et al., 1980); University of Los Angeles, Loneliness scale version 3 (UCLA v3; 20 items) (Russell, 1996); Short Scale for Measuring Loneliness (SSL; 3 items; Hughes et al., 2004); De Jong Gierveld Loneliness Scale (DJLS,11 items) (de Jong-Gierveld & Kamphuls, 1985); Short De Jong Gierveld Loneliness Scale (DJLS, 6 items) (de Jong-Gierveld & Tilburg, 2006).

Data Extraction Form:

Data extracted included: authors, study design, sample size, baseline characteristics, country and culture categorized following the guidelines of Hofstede et al. (2010), loneliness as primary or secondary outcome, characteristics of the intervention (e.g., intervention focus,

delivery mode) (see Supplemental Material for full data extraction form). , intervention format, number of sessions and their length, brief description of the techniques used), type of comparator, other outcomes used, and time points measurements (n, means, and SDs).

p-values and Sample Sizes for the Nine I-I RCTs

The p-values and baseline sample sizes (n) of intervention (n I) and control groups (n C) for the six I-I RCTs are as follows: Mindfulness-based interventions: $p = .008$, $n = 110$ (*n* Mindfulness-Intervention = 34; *n* Social network site-Intervention = 39; *n* Control = 37) (Hussain et al., 2023); $p < .001$, $n = 153$ (*n* Mindfulness-Intervention = 58; *n* Monitor-Intervention = 58; *n C* = 37) (Lindsay et al., 2019). Prosocial behaviors: $p = .002$, $n = 288$ (*n* Giving-Intervention = 90; *n* Keeping-Intervention = 99; n C = 99) (Lanser & Eisenberger, 2023a); $p < .001$, $n = 286$ (*n* Thank-Intervention = 105; *n* Reflect-Intervention = 85; *n C* = 96) (Lanser & Eisenberger, 2023b). Nature-exposure (Razani et al., 2018): $p < .01$, $n = 78$ (*n* I = 50; *n* C= 28). Virtual interaction with strangers (Yang et al., 2022): $p < .01$, $n = 90$ (*n* I = 45; *n* C = 45).

Risk of Bias

Overall, the quality of the 25 studies (s) raised concerns mainly due to unreported pre-registered analysis plans. Specifically, 80% (s = 20) of the studies showed reporting bias or, at least, some concerns due to the lack of either a pre-registered trial protocol with data analysis plan included, or a pre-registered statistical analysis plan. The second reason is related to the randomization process. Particularly, 40% (s = 10) of the studies led to some concerns as they do not report whether allocation was concealed from participants, and one study (Morgado et al., 2023) showed high risk because the baseline loneliness severity significantly differed between groups. Regarding missing data management, 56% (s = 14) of

the studies reported less than 5% of attrition or no attrition at all, and one study (Thimmapuram et al., 2021) showed high risk of bias due to unreported methods to handle missing values. In terms of measuring the outcome, 16% of the studies (s = 4) (Mahmoudpour et al., 2021; Morgado et al., 2023; Myhre et al., 2017; Ristolainen et al., 2020) showed high risk due to unreported blinding of participants which may have influenced their answers to outcome questionnaires due to awareness of intervention received. As for the effects of assignment to intervention, 40% of the studies (s = 10) adhered to the intention-to-treat principle. Finally, 16% of the studies (s = 4) had a high-risk overall assessment (Morgado et al., 2023; Myhre et al., 2017; Ristolainen et al., 2020; Thimmapuram et al., 2021).

Risk of Publication

Publication bias was assessed in the full set of studies, and in the subset of 11 RCTs after excluding the extreme study and the high RoB studies.

In the first case, the Begg's rank correlation test was significant (estimate = 124.00, SE = 33.12, $z = 3.74$, $p < .001$). Egger's test was also significant (intercept = 6.10, SE = 1.22, $t = 4.99_{(df = 19)}$, $p < .001$). After the trim-and-fill method, Begg's and Egger's tests reported a non-significant bias estimate of 29.00 (SE = 50.55), $z = .57$, $p = .566$, with no significant asymmetry (intercept = .64 (SE = 1.86), $t = .35_{(df = 26)}$, $p = .733$).

In the second case, Begg's rank correlation test showed a significant bias estimate of 80.00 (SE = 22.21), $z = 3.60$, $p < .001$. Egger's test also indicated a significant bias estimate of 5.90 (SE = 1.44), $t = 4.04_{(df = 14)}$, $p < .001$. After the trim-and-fill method, Begg's and Egger's tests reported a no significant bias estimate of 13.00 (SE = 35.38), $z = .37$, $p = .713$, with no significant asymmetry (intercept = .40 (SE = 2.08), $t = .19_{(df = 20)}$, $p = .848$).

Table S1

Pre-settled loneliness cut-off inclusion criterion reported by three of the included studies

Study ID	Cut-off	Loneliness outcome
Aydın & Kutlu, 2021	≥ 32	UCLA-LS (Russell et al., 1978)
Bruehlman-Senecal et al., 2020	≥ 21	UCLA-8 (Hays & DiMatteo, 1987)
Käll et al., 2023	≥ 55	UCLA v3 (Russell, 1996)

Table S2.

Characteristics of the 25 reviewed studies

Study ID<br><br>Country (Hofstede category)	Design	Sample<br><br>n<br>% women<br>Mean age (SD)<br>Population	Loneliness intervention<br><br>Experimental group<br>*Main active component* (# groups)<br><br>Duration:<br># sessions (length per session); total length (months)<br><br>Component (Single or Multiple)	Control Group (# groups)	Intervention-focus (Person- or Context-focus)<br><br>Delivery-mode (Virtual or In person)<br><br>Intervention-format (Individual or Group)	Follow-up (months)	Loneliness outcome
**Aydin and Kutlu, 2021**<br><br>Turkey (Collectivistic)	RCT<br>Stratified Randomization<br>Single-blind (Participant)<br>Block Method	60<br>78.3%<br>72.56 (± 1.01)<br>Older Adults	*Art therapy* (1)<br><br>x6 (60 - 90 min); length (n/r)<br>Opportunities for interaction and verbal expression.<br><br>Single component	No intervention (1)	Person-focused<br><br>In person<br><br>Group	No	UCLA-LS (Russell et al., 1978)
**Bruehlman-Senecal et al., 2020**<br><br>United States (Individualistic)	RCT<br>Open label<br>Parallel assignment	221<br>59.3%<br>18.68 (.35)<br>College Students	*CBT* (1)<br><br>N/r (n/r); 4 weeks<br>App exercises for cognitive restructuring of negative social experiences and savoring of positive social experience. Based on positive psychology, mindfulness-based self-compassion, and cognitive behavioral skill-building.<br><br>Multiple component	Delayed access (1)	Person-focused<br><br>Virtual<br><br>Individual	No	UCLA-8 (Hays & DiMatteo, 1987)
**Cohen-Mansfield et al., 2018**<br><br>Israel (Collectivistic)	RCT<br>Single-blind (Outcomes assessor)<br>Parallel assignment	74<br>CBT 79.49%<br>CG 82.86%<br><br>CBT 76.6(6.8)<br>CG 79(6.62)<br><br>Older Adults	*CBT* (1)<br><br>x7 individual (n/r)<br>x10 group (n/r); length (n/r)<br>I-SOCIAL CBT-based + environmental barriers; practicing social skills, motivational interviewing.<br><br>Multiple component	No intervention (1)	Person-Context-focused<br><br>In person<br><br>Individual<br>Group	Yes (3)	UCLA-8 (Hays & DiMatteo, 1987)
**Kahlon et al., 2021**	RCT<br>Parallel assignment	240<br>79%	*Social Support* (1)<br><br>x20 (~ less than 10 min; 5 per week); 4 weeks	No intervention (1)	Person-focused<br><br>Virtual	No	SLS (Hughes et al., 2004)<br><br>Short_DJLS (de Jong-Gierveld & Tilburg, 2006)

United States<br>(Individualistic)	Single-Blind<br>(outcome assessors)	IG 69.4(11.5)<br>CG 68.7(12.8)<br>Older adults	Social support through empathic calls. Key question during the call: how the participant is doing, otherwise the call will be unscripted. Interactions were tracked using Redcap.<br><br>Multiple-component		Individual		
**Käll and Andersson, 2023**[c]<br><br>Sweden<br>(Individualistic)	RCT<br>Parallel Assignment<br>Single Blind<br>(Participants)	73<br>71.2%<br>47.2 (17.63)<br>Adults	*CBT* (1)<br><br>x8 (n/r); 8 weeks<br>CBT-based modules. Guided participants received therapist support.<br><br>Single component	Delayed access + guidance on demand (1)	Person-focused<br><br>Virtual<br><br>Individual	Yes (24)	UCLA v3 (Russell, 1996)
**Käll et al., 2021**<br><br>Sweden<br>(Individualistic)	RCT<br>Parallel Assignment<br>Open Label	170<br>75.9%<br>47.5 (16.4)<br>Adults	*CBT* (1)<br>*IPT* (1)<br><br>x9 (n/r); 9 weeks.<br>Both programs -CBT & IPT- were module-based with information about loneliness and social connection supported by trained professionals.<br><br>Single component	Waitlist (1)	Person-focused<br><br>Virtual<br><br>Individual	Yes (4)[b]	UCLA v3 (Russell, 1996)
**Li et al., 2022**<br><br>China<br>(Collectivistic)	RCT	64<br>63.3%<br>65.7 (3.69)<br>Older Adults	*Reminiscence therapy* (1)<br><br>x8 (4 hours); length (n/r)<br>Reminiscence group + Chinese traditional activities (rural environments; needs-based), facilitated by intervention staff.<br><br>Multiple component	No intervention (1)	Person-Context-focused<br><br>In person<br><br>Group	Yes (3)	UCLA-R (Russell et al., 1980)
**Liu, Wegner et al., 2023**<br><br>Germany<br>(Individualistic)	RCT<br>Parallel Assignment<br>Single-Blind	252<br>73%<br>33.93 (11.84)<br>Adults	*Social Support:*<br>Animated video + written messages (1)<br>Animated video (1)<br>Written messages (1)<br><br>x1 (~ 4 min); 1 day<br>Entertainment-Education video about social support, loneliness and COVID-19.<br><br>Multiple component	No intervention (1)	Person-focused<br><br>Virtual<br><br>Individual	No	UCLA-8 (Hays & DiMatteo, 1987)
**Mahmoudpour et al., 2021**<br><br>Iran<br>(Collectivistic)	RCT	83<br>100%<br>32(n/r)<br>Divorced women	*ACT* (1)<br><br>x8 (90 mins); 1 month<br>ACT aiming to increase psychological flexibility through acceptance.	No intervention (1)	Person-focused<br><br>In person<br><br>Group	No	UCLA-LS (Russell et al., 1978)

			Single component				
**Morgado et al., 2021**<br><br>Portugal (Individualistic)	RCT Quasi-experimental	34<br>50%<br>81.97 (8.03)<br>Institutionalized older adults	*Reminiscence therapy (*1)<br><br>x10 (45 mins); length (n/r)<br>Recollection of an experience or fact to which the subject habitually associates pain or pleasure.<br><br>Single component	No intervention (1)	Person-focused<br><br>In person<br><br>Group	No	UCLA-LS (Russell et al., 1978)
**Myhre et al., 2017** [a]<br><br>United States (Individualistic)	RCT	41<br>FB 36 %<br>OD 31%<br>CG 21%<br><br>FB 80 (7.34)<br>OD 78.38 (7.32)<br>CG 79.29 (6.76)<br><br>Community-dwelling older adults	*Digital-Social Support* (1)<br><br>Nr (n/r); 8 weeks<br>Computer skills-FB training sessions, followed by 7 weeks of daily FB use with monitored interactions.<br><br>Multiple-component	Waitlist (1)<br><br><br>Online diary (1)	Person-focused<br><br>Virtual<br><br>Individual	No	UCLA v3 (Russell, 1996)
**Ristolainen et al., 2020**<br><br>Finland (Individualistic)	RCT	392<br>82.9 %<br>76.8 (7.5)<br>Older people living alone	*Social Support* (1)<br><br>x5 (2-3 hours); 6 months<br>to enhance wellbeing by incorporating social support, counseling, and activities, tailored to their needs.<br><br>Multiple component	No Intervention (1)	Person-focused<br><br>In person<br><br>Group	No	UCLA-12 (Junttila et al., 2013)
**Shapira et al., 2021**<br><br>Israel (Individualistic)	RCT	82<br>IG 81%<br>CG78%<br><br>IG 72.1 (5.3)<br>CG 71.7 (6.8)<br>Older Adults	*CBT* (1)<br><br>x7 (60 - 90 min); ~ 4 weeks<br>A CBT-based safe space for social interactions<br><br>Single component	Waitlist (1)	Person-focused<br><br>Virtual<br><br>Group	No	SLS (Hughes et al., 2004)
**Tabrizi et al., 2016**<br><br>Iran (Collectivistic)	RCT Double-blind	140<br>100%<br>47.9 (11.4)<br>Breast cancer survivors	*Social Support* (1)<br><br>x12 (90 min); 12 weeks<br>Leader facilitated expression of fears, existential issues, and coping strategies.<br><br>Multiple component	No intervention (1)	Person-focused<br><br>In person<br><br>Group	Yes (2)	UCLA-R (Russell et al., 1980)
**Thimmapuram et al., 2021**	RCT	155<br>66%	*Meditation* (1)	Delayed access (1)	Person-focused<br><br>Virtual	No	UCLA v3 (Russell, 1996)

United States<br>(Individualistic)		46 (11.03)<br>Physicians	x ~28 Sessions (15 min); 4 weeks<br>Guided audio sessions for morning and bedtime relaxation, emphasizing gentle attention to body and heart, practiced daily.<br><br>Single component		Individual		
**Xiao et al., 2021**<br><br>China<br>(Collectivistic)	RCT	96<br>~26.04%<br>Bb 18.95 (.89)<br>Bj 19.21 (1.02)<br>CG 19.71 (1.77)<br>University Students	*Physical activity* (1)<br><br>*Mindful movement* (1)<br><br>x36 (90 min); 12 weeks<br>Basketball training (Physical activity) or Baduanjin practice (mindful movement) three times per week, including warm-up, exercise, and cooldown.<br><br>Single component	No intervention (1)	Person-focused<br><br>Virtual<br><br>Individual	Yes (2)	UCLA-8 (Hays & DiMatteo, 1987)
Hussain et al., 2023<br><br>United Kingdom<br>(Individualistic)	RCT<br>Single-Blind	110<br>79%<br>20.97(4.83)<br>College Students	*Mindfulness* (1)<br><br>*SNS* (1)<br><br>Nr (n/r); 4 weeks.<br>Mindfulness-based instruction set for addictive urges; SNS intervention involved providing advice about SNS management. Happier life intervention: lifestyle advice.<br><br>Single component	Happier life intervention (1)	Person-focused<br><br>Virtual<br><br>Individual	Yes (0,5)	UCLA-R-11 items (Lee & Cagle, 2017)
Lanser & Eisenberger, 2023**<br><br>United States<br>(Individualistic)	RCT	300<br>79.7%<br>20.08(n/r)<br>Undergraduate students	*Prosocial behavior-Giving* (1)<br><br>*Prosocial behaviour-Keeping* (1)<br><br>x 1 (task done only once); length (n/r)<br>Participants completed tasks online: giving gift cards or keeping one for themselves.<br>Logo viewing: answering questions about different logos.<br><br>Single component	Logo viewing (1)	Person-focused<br><br>Virtual<br><br>Individual	No	UCLA v3 (Russell, 1996)
Lanser & Eisenberger, 2023**<br><br>United States<br>(Individualistic)	RCT	300<br>77.1%<br>20.50 (n/r)<br>Undergraduate students	*Prosocial behavior-Gratitude* (1)<br><br>*Prosocial behaviour-Reflection* (1)<br><br>x1 (task done only once); length (n/r)	TV-show writing (1)	Person-focused<br><br>Virtual<br><br>Individual	No	UCLA v3 (Russell, 1996)

			Participants completed tasks online: expressing gratitude to support figure or reflecting on support.<br>TV show writing: writing about a favorite TV show.<br><br>Single component				
Lindsay et al., 2019<br><br>United States (Individualistic)	RCT<br>Parallel assignment<br>Double-blind	153<br>67.32%<br>32.42 (13.68)<br>Young adults	*Mindfulness (1)*<br><br>x 14 sessions (20 min); 2 weeks<br>Participants learned:<br>MA: concentration (focus on present-moment experiences); sensory clarity (monitoring) + Equanimity (acceptance and openness to experiences)<br>MO: Concentration and clarity. No instruction on acceptance.<br><br>Single component	Coping control (1)	Person-focused<br><br>Virtual<br><br>Individual	No	UCLA v3 (Russell, 1996)
Liu et al., 2023<br><br>Taiwan (Collectivistic)	RCT<br>Parallel Assignment<br>Block Randomization<br>Single-Blind (Participants)	100<br>IG 74%<br>CG 70%<br><br>IG 72.90 (4.45)<br>CG 72.78 (±4.52)<br><br>Older Adults	*Digital-Social Support* (1)<br><br>x60 (1 hour); 12-weeks<br>Bidirectional remote interaction program that included daily interactive online group static (creative arts, music appreciation) and dynamic (aerobics, mouth exercises, acupressure, balance and stretching) sessions.<br>Unidirectional: only watching YouTube courses.<br><br>Multiple component	YouTube-interaction (1)	Person-focused<br><br>Virtual<br><br>Group	No	UCLA v3 (Russell, 1996)
Razani et al., 2018<br><br>United States (Individualistic)	RCT<br>Parallel Assignment<br>Open Label	78<br>87%<br>38 (n/r)<br>Child-parent pairs	*Nature exposure* (1)<br><br>x3 (n/r); 3 weeks<br>Both groups received pediatric counseling, park prescription (outdoor activities promotion), pedometer, and journals. The IG additionally received specific guidance and support from a team expert on park-trails.<br><br>Single component	Independent Park prescription (1)	Context-focused<br><br>In person<br><br>Individual	Yes (3)	UCLA-R (Russell et al., 1980)
Van Orden et al., 2022	RCT<br>Parallel Assignment<br>Open Label	291<br>75.3%<br>72(9.07)<br>Older Adults	*Volunteering* (1)<br><br>N/r (n/r); 12 months	Life review (1)	Person-focused<br><br>In person	No	SSL (Hughes et al., 2004)

United States<br>(Individualistic)			Program connects participants to community volunteer roles, emphasizing skill matching and support.<br>Life review: self-guided life review writing exercises.<br><br>Single component		Individual		
Yang et al., 2023<br><br>Taiwan<br>(Collectivistic)	RCT<br>Parallel Assignments<br>Single Blind<br>(Participants)	90<br>64.04%<br>IG 68.07 (6.68)<br>CG 69.00 (6.04)<br>Older Adults	*Virtual interaction with strangers* (1)<br><br>x40 (n/r); 8 weeks<br>Online intervention via LINE, noon to evening, includes greetings, interactive courses, videos, music, fostering engagement and learning.<br>Non-online interactive intervention receives messages without interactive courses.<br><br>Multiple component	Non-online interactive group (1)	Person-focused<br><br>Virtual<br><br>Individual	No	UCLA v3 (Russell, 1996)
Zhang et al., 2023<br><br>United States<br>(Individualistic)	RCT	132<br>62.9%<br>24.4 (10.1)<br>University Students	*Art therapy* (2)<br><br>x4 (15 min); 4 days<br>Daily online writing expressiveness tasks with imagined interaction. Two types of writing, each tested in different IGs: Rehearsal (anticipated stressor) and replay expressive writing (past mental health stressor) were compared to an active control group (hobbies writing)<br><br>Single component	Hobbies-writing (1)	Person-focused<br><br>Virtual<br><br>Individual	Yes (1)	SLS (Hughes et al., 2004)

Bold indicates I-C design-based studies selected in the meta-analysis (s = 16)
Single-blinding procedures are indicated in parenthesis.
[a] Only one comparison included in the meta-analysis.
[b] Excluded for the follow-up analysis as follow-up data was collected for the experimental group only.
[C] Data from the intervention was also retrieved from Käll et al., 2020a; 2020b.
** Reported in the same publication.
Note: in the presence of more than 2 groups, the acronyms of CG and IG were replaced by the intervention acronym to simplify the table.
ACG = Active Control Group; ACT = Acceptance and Commitment Therapy; Bb = Basketball practice; Bj = Baduanjin practice; CBT = Cognitive Behavioral Therapy; CG = Control Group; FB = Facebook; IG = Intervention Group; IPT = Internet-based Interpersonal Therapy; MA = Monitor + Accept; MO = Monitor Only; N/r = Number of sessions not reported ; n/r = no reported; OD = Online Diary; RCT= Randomized Controlled Trial; SLS = Short Loneliness Scale; SNS = Social Networking Sites; UCLA-LS = University of California, Los Angeles Loneliness Scale; UCLA-R = University of California, Los Angeles Loneliness Scale, the revised version; University of California, Los Angeles Loneliness Scale v3 = University of California, Los Angeles Loneliness Scale version 3; UCLA-8 = 8-item University of California, Los Angeles Loneliness Scale; UCLA-11 = 11-item University of California, Los Angeles Loneliness Scale; UCLA-12 = 12-item University of California, Los Angeles Loneliness Scale

Table S3.

Sensitivity analysis and overall effects and heterogeneity of experimental interventions vs controls on loneliness at post-intervention.

**Overall effects**							***Heterogeneity***			
**Random Effect Model (HK)**	**s**	**k**	***g***	**95% CI**	***p-value***	**NNT**	***Q* (df) and *I*²** [95% CI for *I*²]		**τ²**	**[95% CI]**
Post-intervention							*Post-intervention*			
All I-C RCTs (overall)	16	21	.65	[.05; 1.26]	.037	2.82	$Q_{(20)}$ = 216.75, *p* < .0001	I^2 = 91%, [87%; 93%]	.40	[.62; 2.55]
Removing mixed intervention-format[a]	15	20	.67	[.03;1.32]	.041	2.72	$Q_{(19)}$ = 216.73, *p* < .001	I^2 = 91%, [88%; 94%]	.42	[.64; 2.75]
Removing Audio-Only and Phone-Only Interventions[b]	14	18	.76	[.00; 1.52]	.050	2.44	$Q_{(17)}$ = 213.42, *p* < .001	I^2 = 92%, [89%; 94%]	.54	[.75; 3.46]
Removing studies with high risk of bias (RoB)[c]	12	17	.81	[.03; 1.58]	.043	2.32	$Q_{(16)}$ = 192.92, *p* < .001,	I^2 = 92%, [88%; 94%]	.48	[.72; 3.42]
Removing study with the highest weight[d]	15	20	.46	[.19; .73]	.002	3.92	$Q_{(19)}$ = 116.73, *p* < .001,	I^2 = 84%, [76%; 89%]	.20	[.13; .63]
Removing highest weighted study & studies high RoB[e]	11	16	.55	[.22; .88]	.003	3.29	$Q_{(15)}$ = 95.41, *p* < .001,	I^2 = 84%, [76%; 90%]	.22	[.13; 0.78]
Follow-up										
Follow-up: All I-C RCTs[f]	6	8	1.91	[-1.87; 5.69]	.251	1.22	$Q_{(5)}$ = 113.27, *p* < .001,	I^2 = 95.6%, [93%; 97%]	1.88	[2.19; 40.01]
Follow-up: Intervention Format Sensitivity	4	5	2.36	[-2.67; 7.40]	.262	1.10	$Q_{(d4)}$ = 106.55, *p* < .001,	I^2 = 96%, [94%; 98%]	2.43	[2.52; 70.43]
Follow-up[g]	3	4	.74	[-.08; 1.56]	.067	2.52	$Q_{(4)}$ = 22.05, *p* < .001,	I^2 = 81.9%, [58%; 92%]	.32	[.08; 3.40]

[a] Sensitivity analysis removing mixed intervention-format data (Cohen-Mansfield et al., 2018)
[b] Sensitivity analysis removing delivery-mode data of interventions lacking (Kahlon et al., 2021a; Kahlon et al., 2021b; Thimmapuram et al., 2021)
[c] Sensitivity analysis removing high RoB studies (Morgado et al., 2023; Myhre et al., 2017; Ristolainen et al., 2020; Thimmapuram et al., 2021)
[d] Sensitivity analysis removing the most weighted study (Lit et al., 2021)
[e] Sensitivity analysis removing the most weighted study & high RoB studies
[f] Follow-up analyses for sensitivity analysis delivery-mode and high RoB are included here as studies removed did not have follow-up data.
[g] Similarly, follow-up analysis for most weighted study and high RoB are included here.
df = degrees of freedom; g = Hedge's g ES; I^2 = Heterogeneity; I-C = Intervention Control design; k = number of effect sizes; NNT = number needed-to-treat; Q = Q test; RoB = Risk of Bias (based on the tool from Cochrane); s = number of studies; τ² = tau square. *$p < 0.05$; ** $p < 0.01$

Table S4.

Effects of interventions tackling loneliness compared to controls: subgroup analyses (k = 16)

Subgroups comparisons	Within groups							Subgroup differences
	k	*g*	95% CI	NNT	*Q*	I^2 (%)	τ^2	$Q_{(df)*}$, *p value*
Hofstede category								*Q* = 9.92, *p < .001*
Collectivism-based	5	1.21	[.47; 1.95]	1.64	16.99	76.5%	.27	
Individualism-based	11	.29	[.01; .56]	6.24	39.13	74.4%	.098	
Intervention focus								*Q* = .89, *p* .346
Person	15	.57	[.22; .92]	3.18	95.36	85.3%	.23	
Context	1	.30	[-.16; .76]	5.88	.00	---	---	
Delivery mode								*Q* = .73, *p* .391
In-person	4	.81	[-.28; 1.89]	2.31	12.15	75.3%	.23	
Virtual	12	.48	[.10; .86]	3.76	77.61	85.8%	.22	
Intervention format								*Q* = 1.39, *p* .239
Group	5	.82	[.10; 1.54]	2.29	13.01	69.2%	.17	
Individual	11	.44	[.03; .85]	4.06	72.32	86.2%	.21	
Loneliness as the primary outcome								*Q* = 16.80, *p < .001*
Yes	2	1.49	[-1.35; 4.33]	4.38	1.25	19.7%	.02	
No	14	.41	[.11; .71]	1.41	60.14	78.4%	---	
Active component								*Q* = 36.99, *p < .001*
Art therapy	1	.91	[.38; 1.45]	2.08	.00	---	---	
CBT (ACT included)	6	.73	[.07; 1.40]	2.52	29.22	82.9%	.28	
IPT	1	.45	[-.02; .91]	4.02	.00	---	---	
Physical activity	1	1.72	[1.15; 2.30]	1.29	.00	---	---	
Mindful movement	1	1.28	[.74; 1.81]	1.58	.00	---	---	
Social support	6	.12	[-.21; .45]	15.26	17.38	71.2%	.07	

[a] Meta-regressions exploring subgroup differences

Hofstede category	R^2 = 44.85%	$QE_{(df = 14)}$ = 56.12, *p* = .004	I^2 = 75.05%, τ^2 = .12 (SE = .06)
Loneliness as the primary outcome	R^2 = 38.72%	$QE_{(df = 14)}$ = 61.38, *p* <.001	I^2 = 77.19%, τ^2 = .13 (SE = .07)
[b] Active component: CBT and social support	R^2 = 3.15%	$QE_{(df = 10)}$ = 46.60, *p* <.001	I^2 = 78.54%, τ^2 = .13 (SE = .08)

Note: Data from the studies included in the sensitivity analysis removing the extreme study (Lit et al., 2021) & high RoB2 studies (Morgado et al., 2023; Myhre et al., 2017; Ristolainen et al., 2020; Thimmapuram et al., 2021).

[a] Meta-regressions were conducted only for subgroups with significant differences

[b] Significant Q-tests of categorical subgroups were interrogated with meta-regressions for the largest subgroups: CBT and social support

*Degrees of freedom were 1 per each comparison except the active component comparison that involved 5 df.

ACT = Acceptance and Commitment Therapy; CBT = Cognitive and Behavioral Therapy; df = degrees of freedom; g = Hedge's g ES; I^2 = Heterogeneity; IPT = Internet-based Interpersonal Therapy; k = number of effect sizes; NNT = number-needed-to-treat; Q = Cochran's Q statistic for heterogeneity (within or between groups); R^2 = amount of heterogeneity accounted for; RoB = Risk of Bias (based on the tool from Cochrane); τ^2 = tau squared.

**p* < 0.05; ** *p* < 0.01

Table S5

Mixed-effects meta-regression with baseline loneliness severity as a moderator: Average baseline loneliness scores rescaled to UCLA v3

	Average baseline means	Standardized baseline means	Min-score	Max-score	Scale
1	2.44	44.40	0	6	Short_DJLS (Gierveld & Tilburg, 2006)
2	6.50	54.90	3	9	SLS (Hughes et al., 2004)
3	5.75	47.50	3	9	SLS (Hughes et al., 2004)
4	18.90	47.23	8	32	UCLA-8 (Hays & DiMatteo, 1987)
5	16.10	40.25	8	32	UCLA-8 (Hays & DiMatteo, 1987)
6	16.50	41.25	8	32	UCLA-8 (Hays & DiMatteo, 1987)
7	16.20	40.50	8	32	UCLA-8 (Hays & DiMatteo, 1987)
8	63.76	63.76	20	80	UCLA-LS (Russell et al., 1978)
9	54.87	54.87	20	80	UCLA-LS (Russell et al., 1978)
10	34.49	34.49	20	80	UCLA-R (Russell et al., 1980)
11	59.11	59.11	20	80	UCLA v3 (Russell, 1996)
12	58.40	58.39	20	80	UCLA v3 (Russell, 1996)
13	58.82	58.82	20	80	UCLA v3 (Russell, 1996)

Note: The rescaling process was conducted using the formula: (score−min score) / (max score−min score)*60+20(score−min score) / (max score−min score)*60+20. Each score was proportionally scaled to a 60-point range, then adjusted to match the 20-80 scale of the UCLA v3. With this standardization process the baseline loneliness severity was treated as a continuous moderator, and consistent interpretations of its association with interventions efficacy were made. For the complete R script please go to the following link: https://osf.io/d3rak/?view_only=3dd83d8426884b6885f547e78953e853

References

de Jong-Gierveld, J., & Kamphuls, F. (1985). The development of a Rasch-type loneliness scale. *Applied psychological measurement, 9*(3), 289-299. https://doi.org/10.1177/014662168500900307

de Jong-Gierveld, J., & van Tilburg, T. G. (2006). A 6-item scale for overall, emotional, and social loneliness: Confirmatory tests on survey data. R*esearch on aging, 28*(5), 582-598. https://doi.org/10.1177/0164027506289723

Hays, R. D., & DiMatteo, M. R. (1987). A Short-Form Measure of Loneliness. *Journal of Personality Assessment*, *51*(1), 69–81. https://doi.org/10.1207/s15327752jpa5101_6

Junttila, N., Ahlqvist-Björkroth, S., Aromaa, M., Rautava, P., Piha, J., & Räihä, H. (2015). Intercorrelations and developmental pathways of mothers' and fathers' loneliness during pregnancy, infancy and toddlerhood–STEPS study. *Scandinavian Journal of Psychology*, *56*(5), 482-488. https://doi.org/10.1111/sjop.12241

Russell, D. (1996). UCLA Loneliness Scale (Version 3): Reliability, validity, and factor structure. *Journal of personality assessment, 66*(1), 20-40. https://doi.org/10.1207/s15327752jpa6601_2

Russell, D., Peplau, L. A., & Cutrona, C. E. (1980). The revised UCLA Loneliness Scale: concurrent and discriminant validity evidence. *Journal of personality and social psychology, 39*(3), 472. https://doi.org/10.1037/0022-3514.39.3.472

Russell, D., Peplau, L. A., & Ferguson, M. L. (1978). Developing a measure of loneliness. *Journal of personality assessment, 42*(3), 290-294. https://doi.org/10.1207/s15327752jpa4203_11
