# Supplementary material for: Loneliness as a Public Health Challenge: A Systematic Review and Meta-Analysis to Inform Policy and Practice
Source: Eur J Investig Health Psychol Educ. 2025 Jul 11;15(7):131. doi: 10.3390/ejihpe15070131 (PMC12293955; doi:10.3390/ejihpe15070131)
Supplement: Supplementary file 1 [file ejihpe-15-00131-s001.zip › Supplement 2_Risk of Bias overall results, Figures S1 and S2.pdf]

Figure S1

Cochrane Risk of Bias 2.0 assessment tool applied to the 25 included studies in the Systematic Review: Overall results

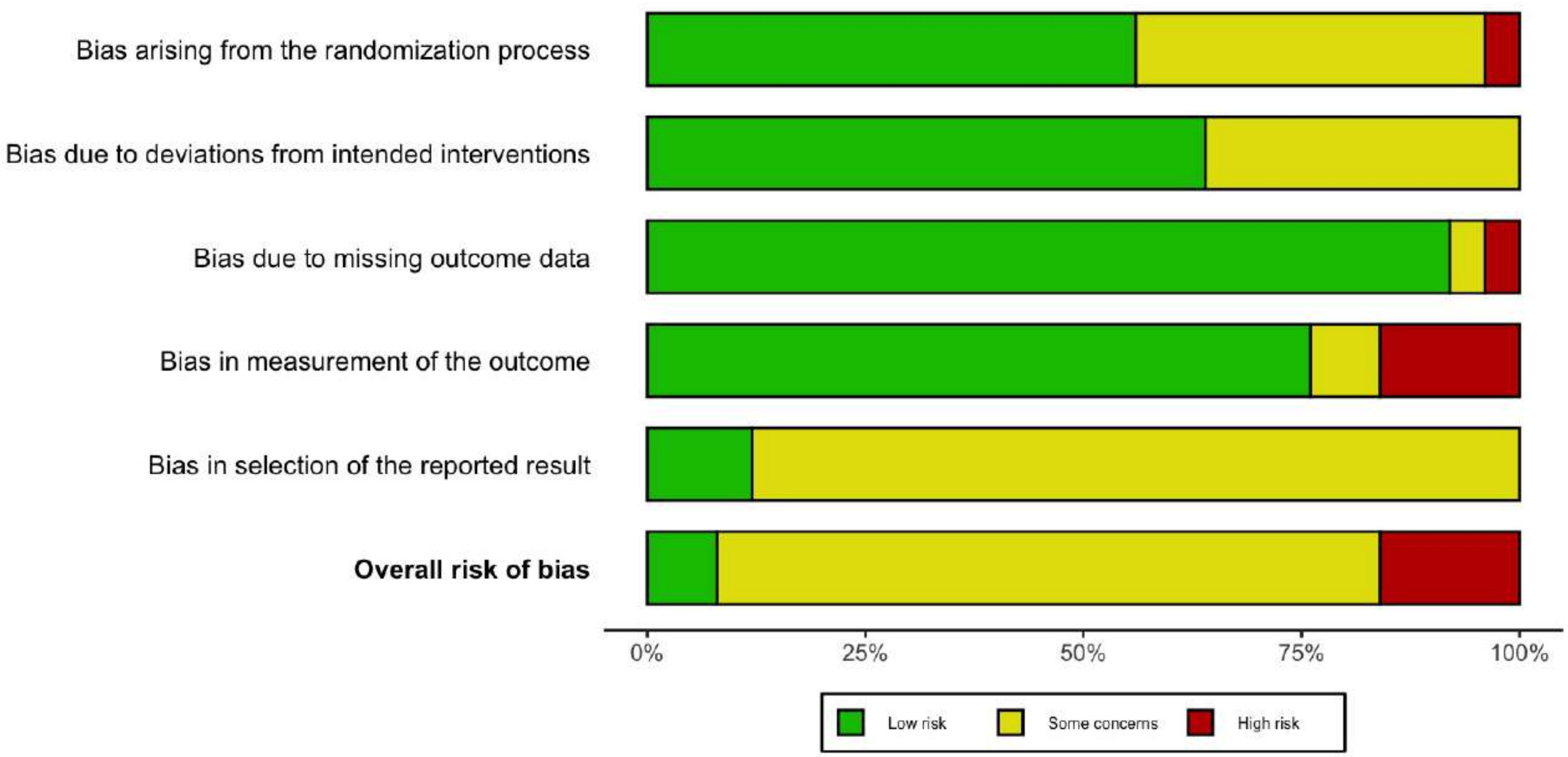

Figure S2

Cochrane Risk of Bias 2.0 assessment tool applied to the 25 included studies in the Systematic Review.

Study	Risk of bias domains: D1	D2	D3	D4	D5	Overall
Aydın and Kutlu, 2021	-	-	+	+	-	-
Bruehlman-Senecal et al., 2020	+	+	+	+	-	-
Cohen-Mansfield et al., 2018	-	-	-	+	-	-
Hussain et al., 2023	-	+	+	+	-	-
Kahlon et al., 2021	+	+	+	+	+	+
Käll and Andersson, 2023	+	+	+	+	-	-
Käll et al., 2021	+	+	+	+	-	-
Lanser & Eisenberger, 2023a	+	+	+	+	-	-
Lanser & Eisenberger, 2023b	+	+	+	+	-	-
Li et al., 2022	+	+	+	+	-	-
Lindsay et al., 2019	+	+	+	+	-	-
Liu et al., 2023	+	-	+	+	-	-
Liu, Wegner et al., 2023	-	-	+	+	-	-
Mahmoudpour et al., 2021	-	-	+	X	-	-
Morgado et al., 2023	X	-	+	X	-	X
Myhre et al., 2017	-	+	+	X	-	X
Razani et al., 2018	+	+	+	-	+	-
Ristolainen et al., 2020	-	+	+	X	-	X
Shapira et al., 2021	+	-	+	+	-	-
Tabrizi et al., 2016	+	+	+	+	-	-
Thimmapuram et al., 2021	-	-	X	+	-	X
Van Orden et al., 2022	+	+	+	+	+	+
Xiao et al., 2021	-	-	+	-	-	-
Yang et al., 2023	+	+	+	+	-	-
Zhang et al., 2023	-	+	+	+	-	-

Domains:
D1: Bias arising from the randomization process.
D2: Bias due to deviations from intended intervention.
D3: Bias due to missing outcome data.
D4: Bias in measurement of the outcome.
D5: Bias in selection of the reported result.

Judgement
X High
- Some concerns
+ Low
