# Supplementary material for: Loneliness as a Public Health Challenge: A Systematic Review and Meta-Analysis to Inform Policy and Practice
Source: Eur J Investig Health Psychol Educ. 2025 Jul 11;15(7):131. doi: 10.3390/ejihpe15070131 (PMC12293955; doi:10.3390/ejihpe15070131)
Supplement: Supplementary file 1 [file ejihpe-15-00131-s001.zip › Supplement 3_Follow-up Forest plot, Figure S3.pdf]

Figure S3

Forest plot of the effect sizes of interventions vs. controls at follow-up (k = 6)

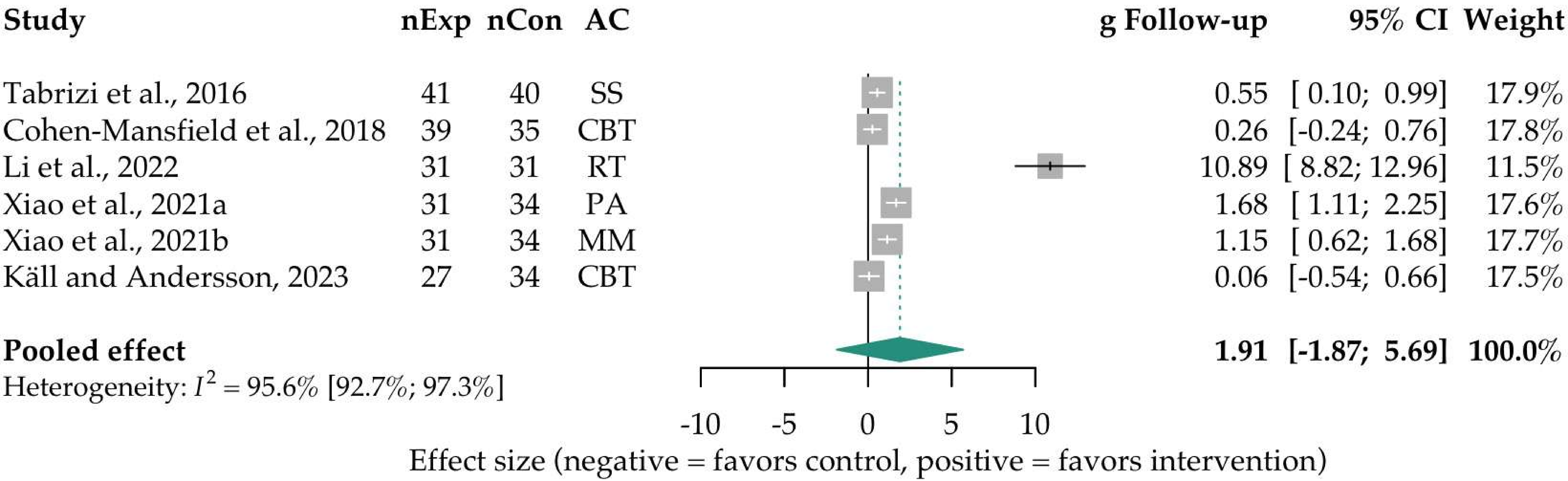

Note. Random-effects model with Hartung–Knapp adjustment for a more accurate standard error.
AC = Main Active Component used in each intervention; CBT = Cognitive Behavioral Therapy; CI = Confidence Interval; g Follow-up = Hedges' g at follow-up; I2 = heterogeneity; MM = Mindful Movement; nExp = Experimental group sample; nCon = Control group sample; PA = Physical Activity; RT = Reminiscence Therapy; SS = Social Support.
