# Supplementary material for: Loneliness as a Public Health Challenge: A Systematic Review and Meta-Analysis to Inform Policy and Practice
Source: Eur J Investig Health Psychol Educ. 2025 Jul 11;15(7):131. doi: 10.3390/ejihpe15070131 (PMC12293955; doi:10.3390/ejihpe15070131)
Supplement: Supplementary file 1 [file ejihpe-15-00131-s001.zip › Supplement 4_Forest plots of the five sensitivity analyses, Figures S4-S8.pdf]

Figure S4

Forest plot of the effect sizes of the interventions vs. controls at post intervention after removing the study with mixed intervention format (k = 20)

Study	nExp	nCon	AC	g Post	95% CI	Weight
Tabrizi et al., 2016	41	40	SS	0.46	[0.02; 0.91]	5.3%
Myhre et al., 2017a	14	14	D_SS	0.47	[-0.28; 1.23]	4.4%
Bruehlman-Senecal et al., 2020	100	121	CBT	0.03	[-0.23; 0.30]	5.6%
Ristolainen et al., 2020	185	207	SS	-0.15	[-0.35; 0.04]	5.8%
Aydın and Kutlu, 2021	30	30	AT	0.91	[0.38; 1.45]	5.0%
Kahlon et al., 2021a	107	119	SS	0.48	[0.22; 0.75]	5.6%
Kahlon et al., 2021b	107	119	SS	0.17	[-0.09; 0.43]	5.7%
Li et al., 2022	31	31	RT	8.81	[7.14; 10.49]	2.1%
Mahmoudpour et al., 2021	15	15	CBT	1.96	[1.07; 2.86]	3.9%
Shapira et al., 2021	64	18	CBT	0.93	[0.39; 1.48]	5.0%
Thimmapuram et al., 2021	40	57	Medt	0.15	[-0.25; 0.56]	5.3%
Xiao et al., 2021a	31	34	PA	1.28	[0.74; 1.81]	5.0%
Xiao et al., 2021b	31	34	MM	1.72	[1.15; 2.30]	4.9%
Käll et al., 2021a	46	27	CBT	0.99	[0.49; 1.49]	5.1%
Käll et al., 2021b	57	27	IPT	0.45	[-0.02; 0.91]	5.2%
Morgado et al., 2023	17	17	RT	0.02	[-0.65; 0.69]	4.6%
Käll and Andersson, 2023	27	34	CBT	0.67	[0.15; 1.19]	5.0%
Liu, Wegner et al., 2023a	63	64	SS	-0.04	[-0.39; 0.31]	5.5%
Liu, Wegner et al., 2023b	61	64	SS	-0.31	[-0.67; 0.04]	5.5%
Liu, Wegner et al., 2023c	64	64	SS	-0.07	[-0.42; 0.27]	5.5%
**Pooled effect**				**0.67**	**[0.03; 1.32]**	**100.0%**

Heterogeneity: $I^2 = 91.2\%$ [87.9%; 93.7%]

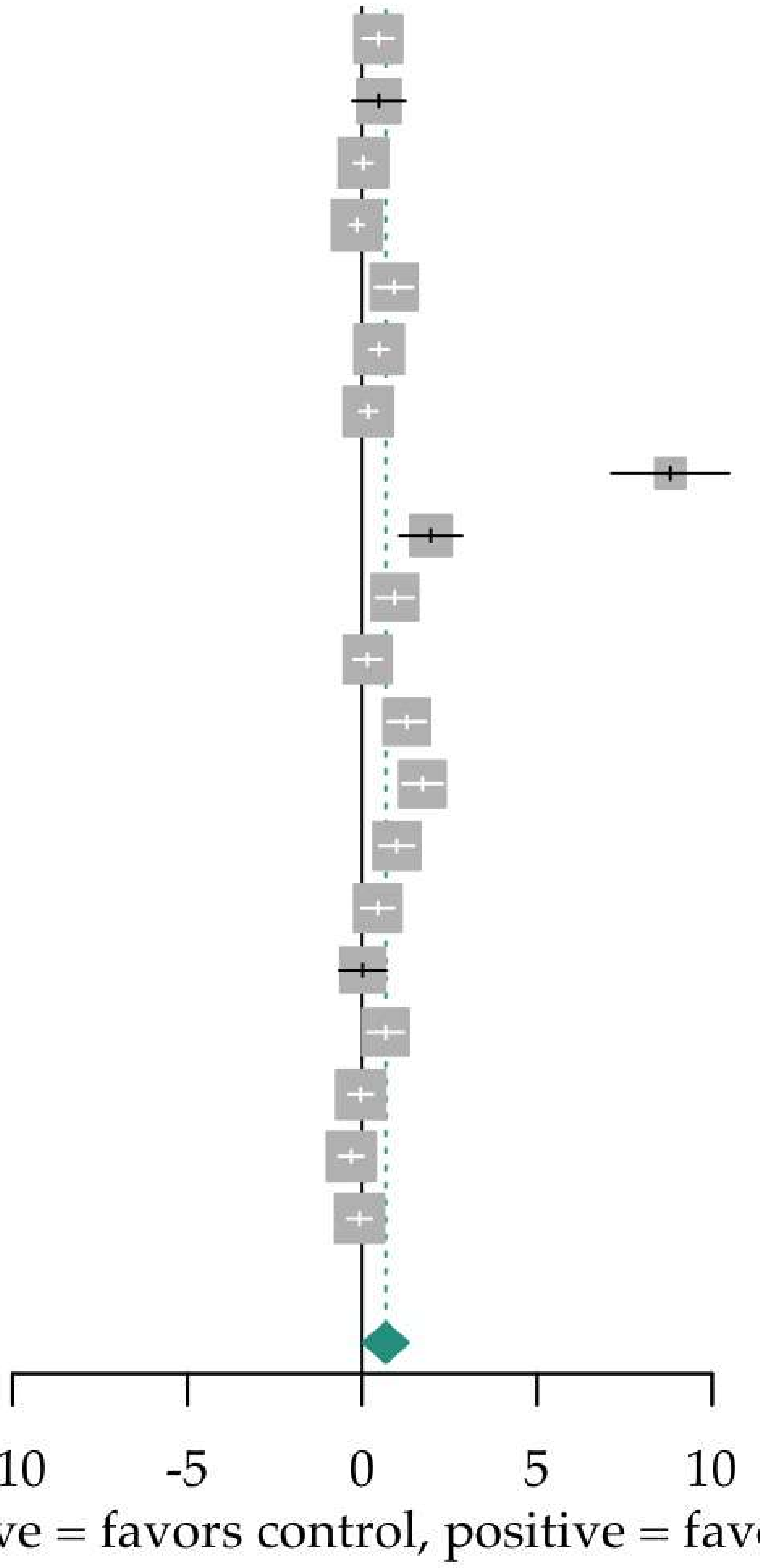

Note. Random-effects model with Hartung–Knapp adjustment for a more accurate standard error.
AC = Main Active Component used in each intervention; AT = Art Therapy; CBT = Cognitive Behavioral Therapy; CI = Confidence Interval; D_SS = Digital Social Support; g Post = Hedges' g at post intervention; I2 = heterogeneity; IPT = Internet-based Interpersonal Therapy; Medt = Meditation; MM = Mindful Movement; nExp = Experimental group sample; nCon = Control group sample; PA = Physical Activity; RT = Reminiscence Therapy; SS = Social Support.

Figure S5

Forest plot of the effect sizes of the interventions vs. controls at post intervention after removing audio-only and phone-only interventions (k = 18)

Study	nExp	nCon	AC	g Post	95% CI	Weight
Tabrizi et al., 2016	41	40	SS	0.46	[0.02; 0.91]	5.9%
Myhre et al., 2017a	14	14	D_SS	0.47	[-0.28; 1.23]	5.1%
Cohen-Mansfield et al., 2018	39	35	CBT	0.30	[-0.16; 0.76]	5.8%
Bruehlman-Senecal et al., 2020	100	121	CBT	0.03	[-0.23; 0.30]	6.2%
Ristolainen et al., 2020	185	207	SS	-0.15	[-0.35; 0.04]	6.3%
Aydın and Kutlu, 2021	30	30	AT	0.91	[0.38; 1.45]	5.7%
Li et al., 2022	31	31	RT	8.81	[7.14; 10.49]	2.7%
Mahmoudpour et al., 2021	15	15	CBT	1.96	[1.07; 2.86]	4.7%
Shapira et al., 2021	64	18	CBT	0.93	[0.39; 1.48]	5.6%
Xiao et al., 2021a	31	34	MM	1.28	[0.74; 1.81]	5.7%
Xiao et al., 2021b	31	34	PA	1.72	[1.15; 2.30]	5.6%
Käll et al., 2021a	46	27	CBT	0.99	[0.49; 1.49]	5.7%
Käll et al., 2021b	57	27	IPT	0.45	[-0.02; 0.91]	5.8%
Morgado et al., 2023	17	17	RT	0.02	[-0.65; 0.69]	5.3%
Käll and Andersson, 2023	27	34	CBT	0.67	[0.15; 1.19]	5.7%
Liu, Wegner et al., 2023a	63	64	SS	-0.04	[-0.39; 0.31]	6.1%
Liu, Wegner et al., 2023b	61	64	SS	-0.31	[-0.67; 0.04]	6.1%
Liu, Wegner et al., 2023c	64	64	SS	-0.07	[-0.42; 0.27]	6.1%
**Pooled effect**				**0.76**	**[0.00; 1.52]**	**100.0%**

Heterogeneity: $I^2 = 92.0\%$ [88.9%; 94.3%]

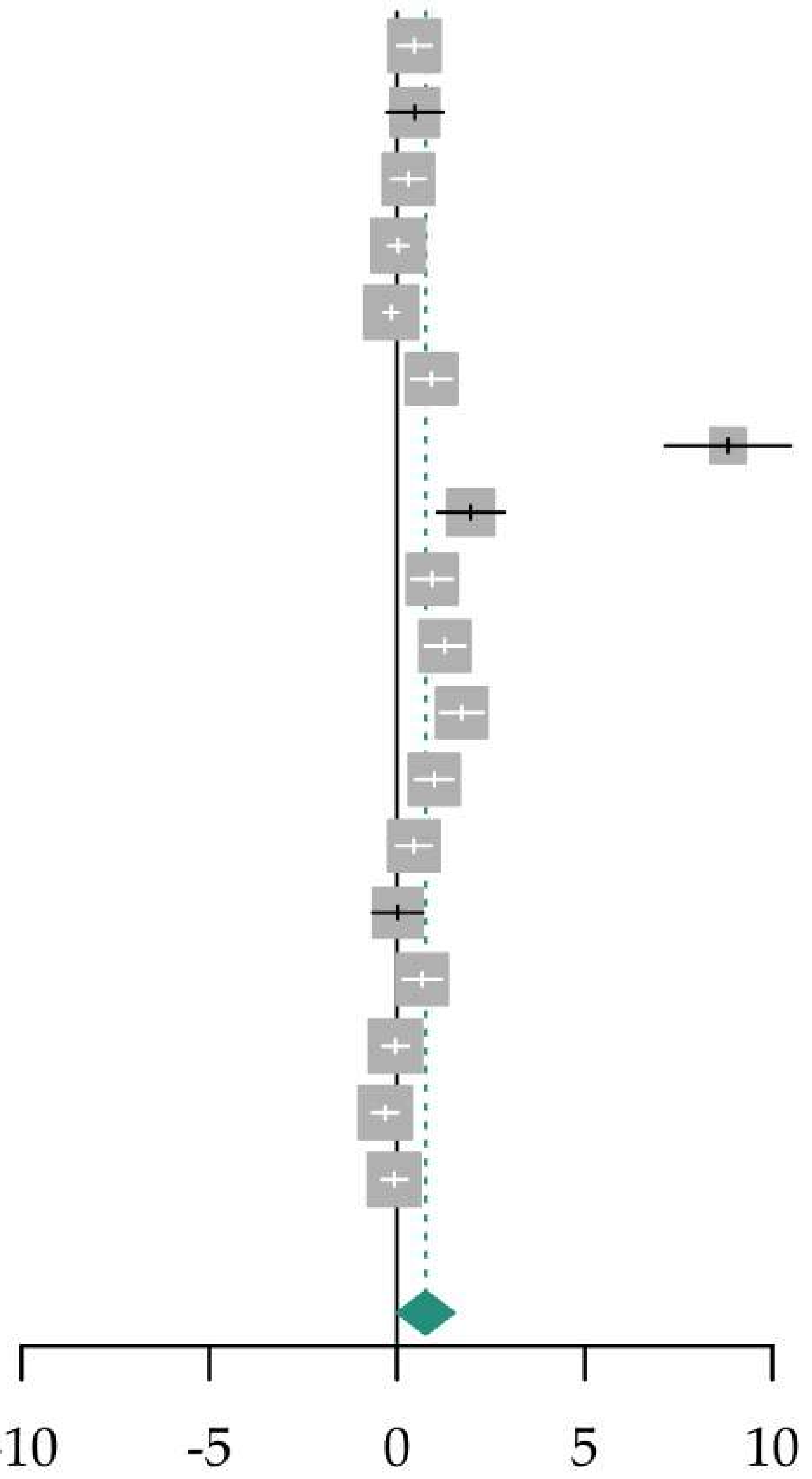

Effect size (negative = favors control, positive = favors intervention)

Note. Random-effects model with Hartung–Knapp adjustment for a more accurate standard error.
AC = Main Active Component used in each intervention; AT = Art Therapy; CBT = Cognitive Behavioral Therapy; CI = Confidence Interval; D_SS = Digital Social Support; g Post = Hedges' g at post intervention; I2 = heterogeneity; IPT = Internet-based Interpersonal Therapy; MM = Mindful Movement; nExp = Experimental group sample; nCon = Control group sample; PA = Physical Activity; RT = Reminiscence Therapy; SS = Social Support.

Figure S6

Forest plot of the effect sizes of the interventions vs. controls at post intervention after removing the high RoB studies (k = 17)

Study	nExp	nCon	AC	g Post	95% CI	Weight
Tabrizi et al., 2016	41	40	SS	0.46	[0.02; 0.91]	6.2%
Cohen-Mansfield et al., 2018	39	35	CBT	0.30	[-0.16; 0.76]	6.1%
Bruehlman-Senecal et al., 2020	100	121	CBT	0.03	[-0.23; 0.30]	6.6%
Aydın and Kutlu, 2021	30	30	AT	0.91	[0.38; 1.45]	5.9%
Kahlon et al., 2021a	107	119	SS	0.48	[0.22; 0.75]	6.6%
Kahlon et al., 2021b	107	119	SS	0.17	[-0.09; 0.43]	6.6%
Li et al., 2022	31	31	RT	8.81	[7.14; 10.49]	2.7%
Mahmoudpour et al., 2021	15	15	CBT	1.96	[1.07; 2.86]	4.8%
Shapira et al., 2021	64	18	CBT	0.93	[0.39; 1.48]	5.9%
Xiao et al., 2021a	31	34	PA	1.28	[0.74; 1.81]	5.9%
Xiao et al., 2021b	31	34	MM	1.72	[1.15; 2.30]	5.8%
Käll et al., 2021a	46	27	CBT	0.99	[0.49; 1.49]	6.0%
Käll et al., 2021b	57	27	IPT	0.45	[-0.02; 0.91]	6.1%
Käll and Andersson, 2023	27	34	CBT	0.67	[0.15; 1.19]	5.9%
Liu, Wegner et al., 2023a	63	64	SS	-0.04	[-0.39; 0.31]	6.4%
Liu, Wegner et al., 2023b	61	64	SS	-0.31	[-0.67; 0.04]	6.4%
Liu, Wegner et al., 2023c	64	64	SS	-0.07	[-0.42; 0.27]	6.4%
**Pooled effect**				**0.81**	**[0.03; 1.58]**	**100.0%**

Heterogeneity: $I^2 = 91.7\%$ [88.3%; 94.1%]

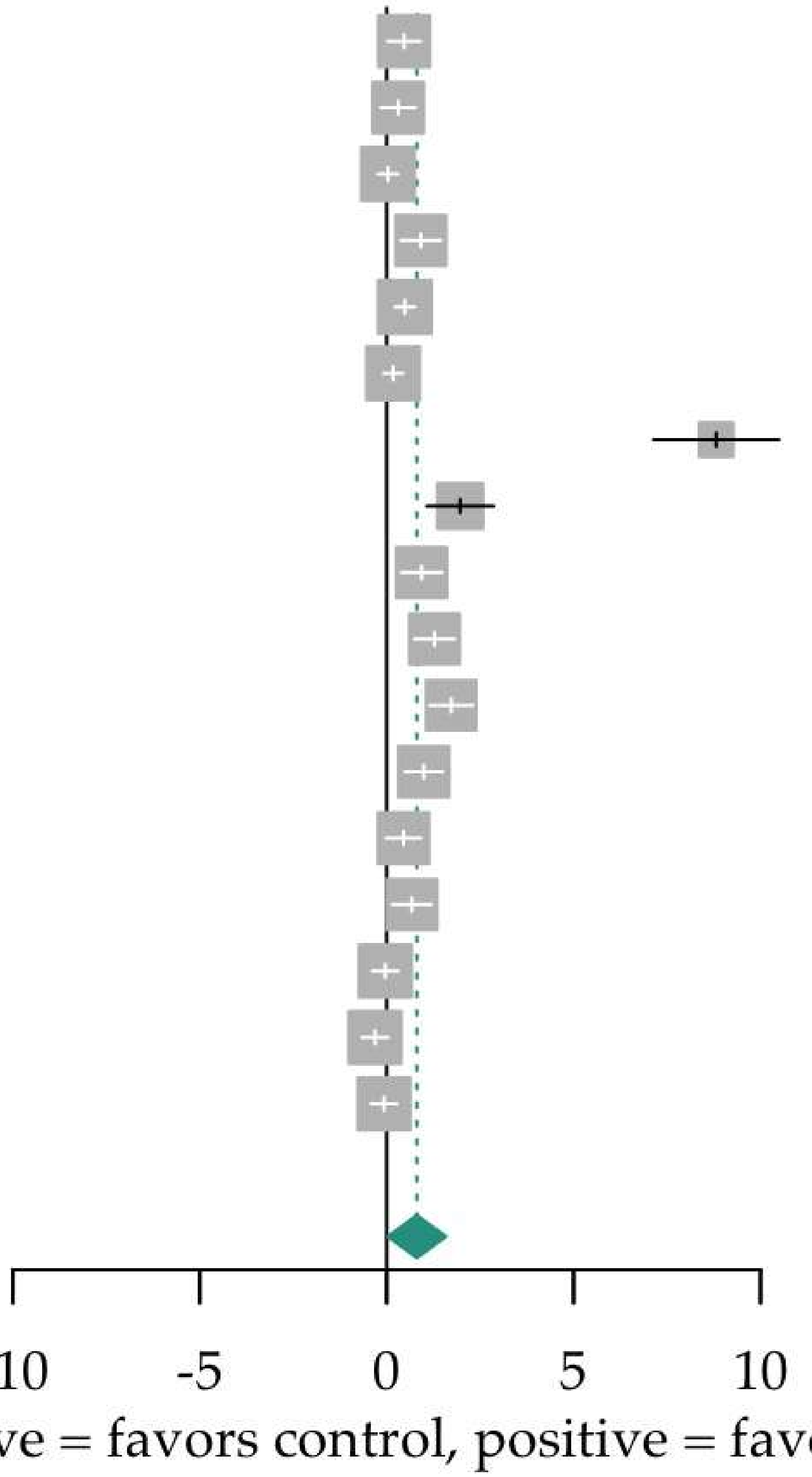

Note. Random-effects model with Hartung–Knapp adjustment for a more accurate standard error.
AC = Main Active Component used in each intervention; AT = Art Therapy; CBT = Cognitive Behavioral Therapy; CI = Confidence Interval; g Post = Hedges' g at post intervention; I2 = heterogeneity; IPT = Internet-based Interpersonal Therapy; MM = Mindful Movement; nExp = Experimental group sample; nCon = Control group sample; PA = Physical Activity; RoB = Risk of Bias; RT = Reminiscence Therapy; SS = Social Support.

Figure S7

Forest plot of the effect sizes of the interventions vs. controls at post intervention after removing the extreme study (k = 20)

Study	nExp	nCon	AC	g Post	95% CI	Weight
Tabrizi et al., 2016	41	40	SS	0.46	[0.02; 0.91]	5.1%
Myhre et al., 2017a	14	14	D_SS	0.47	[-0.28; 1.23]	3.7%
Cohen-Mansfield et al., 2018	39	35	CBT	0.30	[-0.16; 0.76]	5.0%
Bruehlman-Senecal et al., 2020	100	121	CBT	0.03	[-0.23; 0.30]	5.9%
Ristolainen et al., 2020	185	207	SS	-0.15	[-0.35; 0.04]	6.1%
Aydın and Kutlu, 2021	30	30	AT	0.91	[0.38; 1.45]	4.7%
Kahlon et al., 2021a	107	119	SS	0.48	[0.22; 0.75]	5.9%
Kahlon et al., 2021b	107	119	SS	0.17	[-0.09; 0.43]	5.9%
Mahmoudpour et al., 2021	15	15	CBT	1.96	[1.07; 2.86]	3.2%
Shapira et al., 2021	64	18	CBT	0.93	[0.39; 1.48]	4.6%
Thimmapuram et al., 2021	40	57	Medt	0.15	[-0.25; 0.56]	5.3%
Xiao et al., 2021a	31	34	MM	1.28	[0.74; 1.81]	4.7%
Xiao et al., 2021b	31	34	PA	1.72	[1.15; 2.30]	4.5%
Käll et al., 2021a	46	27	CBT	0.99	[0.49; 1.49]	4.8%
Käll et al., 2021b	57	27	IPT	0.45	[-0.02; 0.91]	5.0%
Morgado et al., 2023	17	17	RT	0.02	[-0.65; 0.69]	4.0%
Käll and Andersson, 2023	27	34	CBT	0.67	[0.15; 1.19]	4.8%
Liu, Wegner et al., 2023a	63	64	SS	-0.04	[-0.39; 0.31]	5.6%
Liu, Wegner et al., 2023b	61	64	SS	-0.31	[-0.67; 0.04]	5.5%
Liu, Wegner et al., 2023c	64	64	SS	-0.07	[-0.42; 0.27]	5.6%
**Pooled effect**				**0.46**	**[0.19; 0.73]**	**100.0%**

Heterogeneity: $I^2 = 83.7\%$ [76.0%; 88.9%]

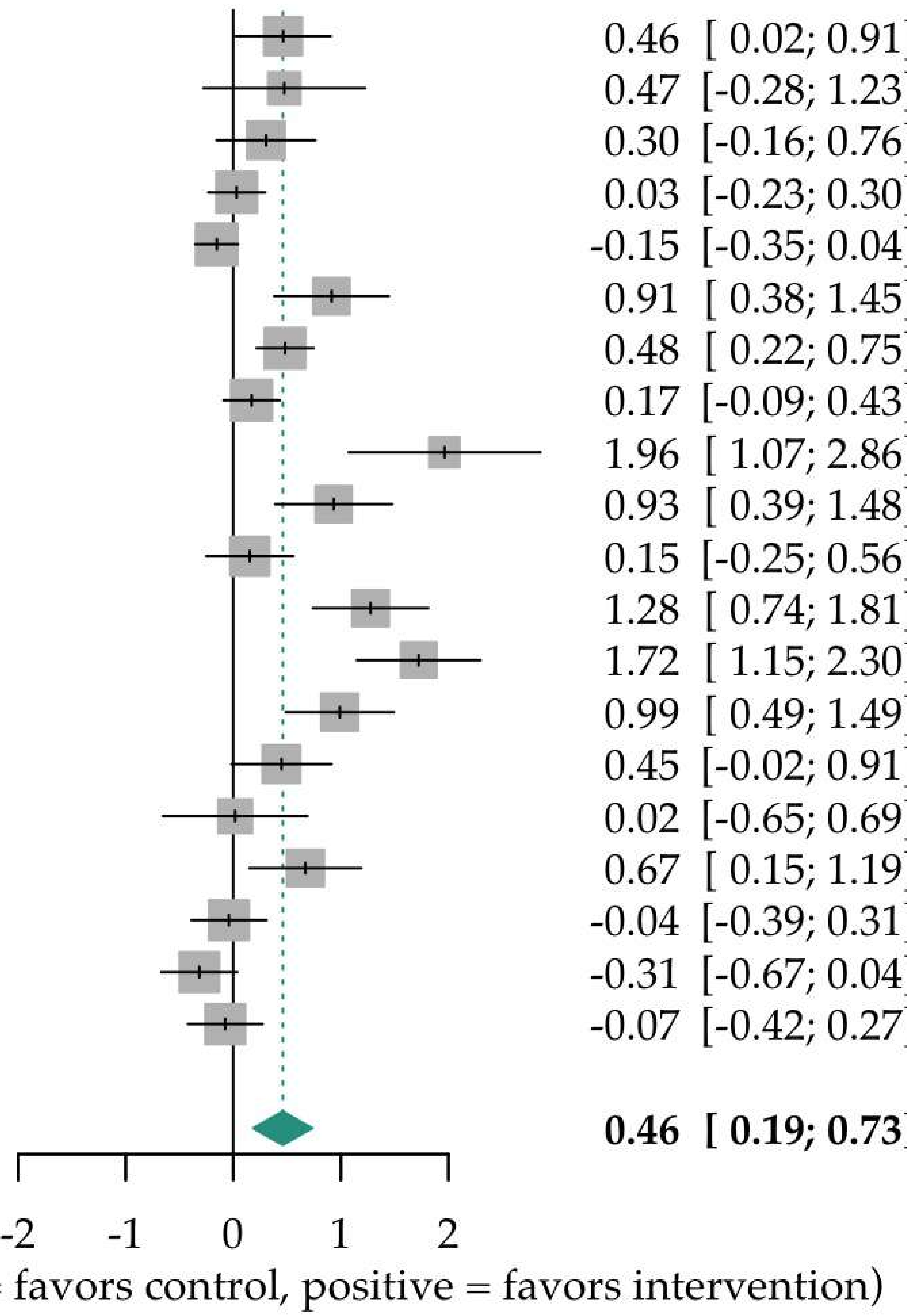

Note. Random-effects model with Hartung–Knapp adjustment for a more accurate standard error.
AC = Main Active Component used in each intervention; AT = Art Therapy; CBT = Cognitive Behavioral Therapy; CI = Confidence Interval; D_SS = Digital Social Support; g Post = Hedges’ g at post intervention; I2 = heterogeneity; IPT = Internet-based Interpersonal Therapy; Medt = Meditation; MM = Mindful Movement; nExp = Experimental group sample; nCon = Control group sample; PA = Physical Activity; RT = Reminiscence Therapy; SS = Social Support.

Figure S8

Forest plot of the effect sizes of the interventions vs. controls at post intervention after removing the extreme study and the high RoB studies (k = 16)

Study	nExp	nCon	AC	g Post	95% CI	Weight
Tabrizi et al., 2016	41	40	SS	0.46	[0.02; 0.91]	6.3%
Cohen-Mansfield et al., 2018	39	35	CBT	0.30	[-0.16; 0.76]	6.3%
Bruehlman-Senecal et al., 2020	100	121	CBT	0.03	[-0.23; 0.30]	7.2%
Aydın and Kutlu, 2021	30	30	AT	0.91	[0.38; 1.45]	5.8%
Kahlon et al., 2021a	107	119	SS	0.48	[0.22; 0.75]	7.2%
Kahlon et al., 2021b	107	119	SS	0.17	[-0.09; 0.43]	7.2%
Mahmoudpour et al., 2021	15	15	CBT	1.96	[1.07; 2.86]	4.0%
Shapira et al., 2021	64	18	CBT	0.93	[0.39; 1.48]	5.8%
Xiao et al., 2021a	31	34	PA	1.28	[0.74; 1.81]	5.8%
Xiao et al., 2021b	31	34	MM	1.72	[1.15; 2.30]	5.6%
Käll et al., 2021a	46	27	CBT	0.99	[0.49; 1.49]	6.0%
Käll et al., 2021b	57	27	IPT	0.45	[-0.02; 0.91]	6.2%
Käll and Andersson, 2023	27	34	CBT	0.67	[0.15; 1.19]	5.9%
Liu, Wegner et al., 2023a	63	64	SS	-0.04	[-0.39; 0.31]	6.8%
Liu, Wegner et al., 2023b	61	64	SS	-0.31	[-0.67; 0.04]	6.8%
Liu, Wegner et al., 2023c	64	64	SS	-0.07	[-0.42; 0.27]	6.8%
**Pooled effect**				**0.55**	**[0.23; 0.88]**	**100.0%**

Heterogeneity: $I^2 = 84.3\%$ [75.8%; 89.8%]

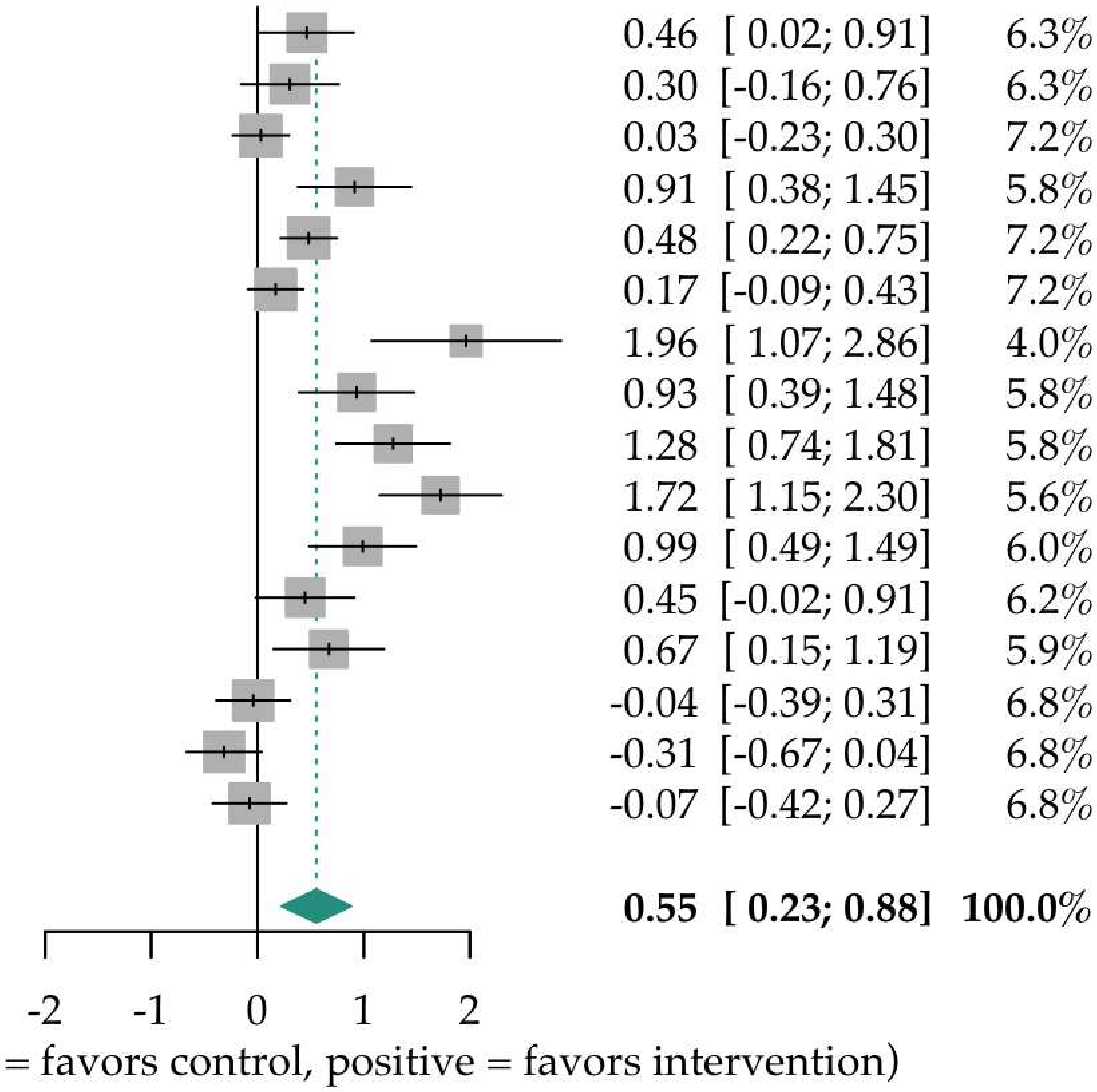

Note. Random-effects model with Hartung–Knapp adjustment for a more accurate standard error.
AC = Main Active Component used in each intervention; AT = Art Therapy; CBT = Cognitive Behavioral Therapy; CI = Confidence Interval; g Post = Hedges' g at post intervention; I2 = heterogeneity; IPT = Internet-based Interpersonal Therapy; MM = Mindful Movement; nExp = Experimental group sample; nCon = Control group sample; PA = Physical Activity; SS = Social Support.
