# Supplementary material for: Loneliness as a Public Health Challenge: A Systematic Review and Meta-Analysis to Inform Policy and Practice
Source: Eur J Investig Health Psychol Educ. 2025 Jul 11;15(7):131. doi: 10.3390/ejihpe15070131 (PMC12293955; doi:10.3390/ejihpe15070131)
Supplement: Supplementary file 1 [file ejihpe-15-00131-s001.zip › Supplement 5_Funnel plots, Figures S9 and S10.pdf]

Figure S9

Funnel plot for effects of interventions tackling loneliness (16 studies)

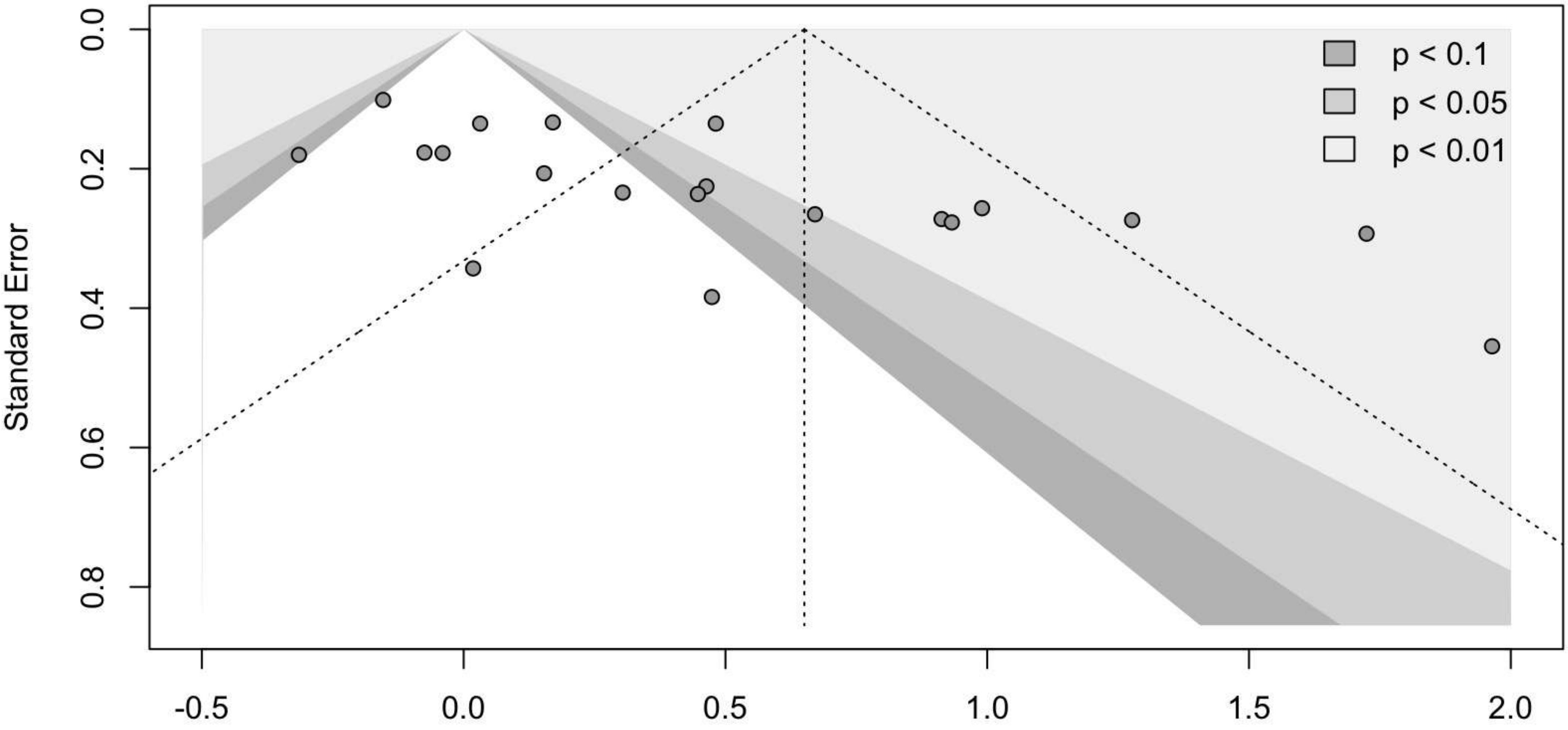

Note. The funnel plot visually assesses publication bias and study heterogeneity for the subset of 11 studies (k = 16). Each gray point represents an individual intervention by its effect size (x axis) and standard error (or precision) (y axis). The shaded areas correspond to different levels of statistical significance (p < .1; p < .05; p < .01) where studies should fall based on their p-values. A concentration of the grey dots in the significant regions (shaded areas) suggests publication bias. The dotted vertical line represents the pooled effect size, and it serves as a reference for comparison.

Figure S10

Funnel plot for effects of interventions tackling loneliness (11 studies)

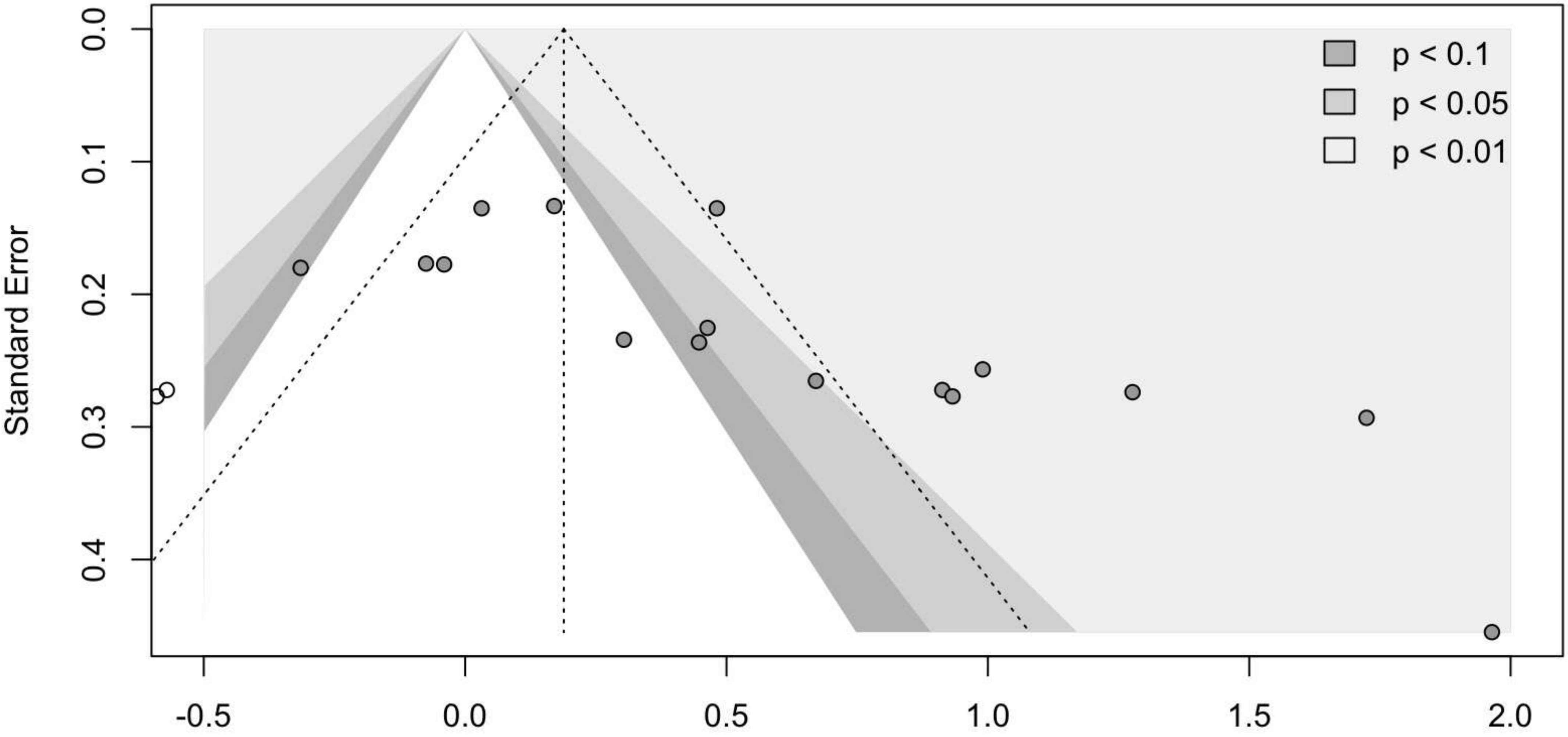

Note. The funnel plot visually assesses publication bias and study heterogeneity for the subset of 11 studies (k = 16). Each gray point represents an individual intervention by its effect size (x axis) and standard error (or precision) (y axis). Each white point represents the added studies after the application of the trim-and-fill method. The shaded areas correspond to different levels of statistical significance ($p < .1$; $p < .05$; $p < .01$) where studies should fall based on their p-values. A concentration of the grey dots in the significant regions (shaded areas) suggests publication bias. The dotted vertical line represents the pooled effect size, and it serves as a reference for comparison.
