# Supplementary material for: Loneliness as a Public Health Challenge: A Systematic Review and Meta-Analysis to Inform Policy and Practice
Source: Eur J Investig Health Psychol Educ. 2025 Jul 11;15(7):131. doi: 10.3390/ejihpe15070131 (PMC12293955; doi:10.3390/ejihpe15070131)
Supplement: Supplementary file 1 [file ejihpe-15-00131-s001.zip › Supplement 6_Forest plots of the subgroup analyses, Figures S11 – S15.pdf]

Figure S11

Forest plot of the effect sizes of the interventions vs. controls at post intervention (k = 16): Hofstede category subgroup analysis

Study	nExp	nCon	AC	g Post	95% CI	Weight
**subgroup = Collectivism**						
Tabrizi et al., 2016	41	40	SS	0.46	[0.02; 0.91]	6.3%
Aydın and Kutlu, 2021	30	30	AT	0.91	[0.38; 1.45]	5.8%
Mahmoudpour et al., 2021	15	15	CBT	1.96	[1.07; 2.86]	4.0%
Xiao et al., 2021a	31	34	PA	1.28	[0.74; 1.81]	5.8%
Xiao et al., 2021b	31	34	MM	1.72	[1.15; 2.30]	5.6%
**Pooled effect**				**1.21**	**[0.47; 1.95]**	**27.7%**
Heterogeneity: $I^2 = 76.5\%$ [42.6%; 90.3%]						
**subgroup = Individualism**						
Cohen-Mansfield et al., 2018	39	35	CBT	0.30	[-0.16; 0.76]	6.3%
Bruehlman-Senecal et al., 2020	100	121	CBT	0.03	[-0.23; 0.30]	7.2%
Kahlon et al., 2021a	107	119	SS	0.48	[0.22; 0.75]	7.2%
Kahlon et al., 2021b	107	119	SS	0.17	[-0.09; 0.43]	7.2%
Shapira et al., 2021	64	18	CBT	0.93	[0.39; 1.48]	5.8%
Käll et al., 2021a	46	27	CBT	0.99	[0.49; 1.49]	6.0%
Käll et al., 2021b	57	27	IPT	0.45	[-0.02; 0.91]	6.2%
Käll and Andersson, 2023	27	34	CBT	0.67	[0.15; 1.19]	5.9%
Liu, Wegner et al., 2023a	63	64	SS	-0.04	[-0.39; 0.31]	6.8%
Liu, Wegner et al., 2023b	61	64	SS	-0.31	[-0.67; 0.04]	6.8%
Liu, Wegner et al., 2023c	64	64	SS	-0.07	[-0.42; 0.27]	6.8%
**Pooled effect**				**0.29**	**[0.01; 0.56]**	**72.3%**
Heterogeneity: $I^2 = 74.4\%$ [53.6%; 85.9%]						
**Pooled effect**				**0.55**	**[0.23; 0.88]**	**100.0%**
Heterogeneity: $I^2 = 84.3\%$ [75.8%; 89.8%]						
Test for subgroup differences: $\chi^2_1 = 9.92$, df = 1 ($p = 0.0016$)						

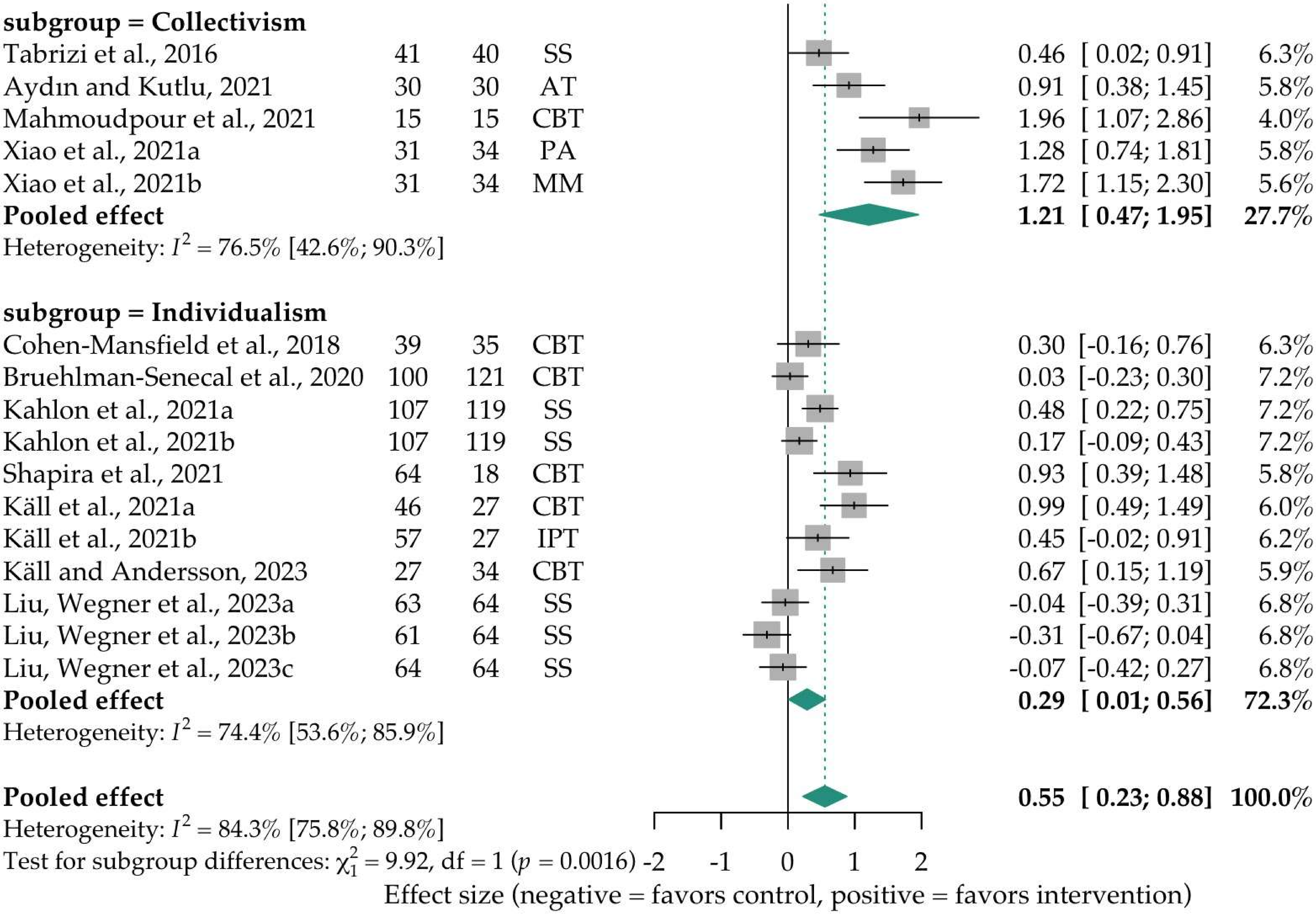

Note. Random-effects model with Hartung–Knapp adjustment for a more accurate standard error.
AC = Main Active Component used in each intervention; AT = Art Therapy; CBT = Cognitive Behavioral Therapy; CI = Confidence Interval; df = degrees of freedom; g Post = Hedges' g at post intervention; I2 = heterogeneity; IPT = Internet-based Interpersonal Therapy; MM = Mindful Movement; nExp = Experimental group sample; nCon = Control group sample; PA = Physical Activity; SS = Social Support

Figure S12

Forest plot of the effect sizes of the interventions vs. controls at post intervention (k = 16): Intervention focus (person- or context-centered) subgroup analysis

Study	nExp	nCon	AC	g Post	95% CI	Weight
**subgroup = Person**						
Tabrizi et al., 2016	41	40	SS	0.46	[0.02; 0.91]	6.3%
Bruehlman-Senecal et al., 2020	100	121	CBT	0.03	[-0.23; 0.30]	7.2%
Aydın and Kutlu, 2021	30	30	AT	0.91	[0.38; 1.45]	5.8%
Kahlon et al., 2021a	107	119	SS	0.48	[0.22; 0.75]	7.2%
Kahlon et al., 2021b	107	119	SS	0.17	[-0.09; 0.43]	7.2%
Mahmoudpour et al., 2021	15	15	CBT	1.96	[1.07; 2.86]	4.0%
Shapira et al., 2021	64	18	CBT	0.93	[0.39; 1.48]	5.8%
Xiao et al., 2021a	31	34	PA	1.28	[0.74; 1.81]	5.8%
Xiao et al., 2021b	31	34	MM	1.72	[1.15; 2.30]	5.6%
Käll et al., 2021a	46	27	CBT	0.99	[0.49; 1.49]	6.0%
Käll et al., 2021b	57	27	IPT	0.45	[-0.02; 0.91]	6.2%
Käll and Andersson, 2023	27	34	CBT	0.67	[0.15; 1.19]	5.9%
Liu, Wegner et al., 2023a	63	64	SS	-0.04	[-0.39; 0.31]	6.8%
Liu, Wegner et al., 2023b	61	64	SS	-0.31	[-0.67; 0.04]	6.8%
Liu, Wegner et al., 2023c	64	64	SS	-0.07	[-0.42; 0.27]	6.8%
**Pooled effect**				**0.57**	**[0.22; 0.92]**	**93.7%**
Heterogeneity: $I^2 = 85.3\%$ [77.3%; 90.5%]						
**subgroup = PersonContext**						
Cohen-Mansfield et al., 2018	39	35	CBT	0.30	[-0.16; 0.76]	6.3%
**Pooled effect**				**0.55**	**[0.23; 0.88]**	**100.0%**
Heterogeneity: $I^2 = 84.3\%$ [75.8%; 89.8%]						
Test for subgroup differences: $\chi^2_1 = 0.89$, df = 1 ($p = 0.3461$)						

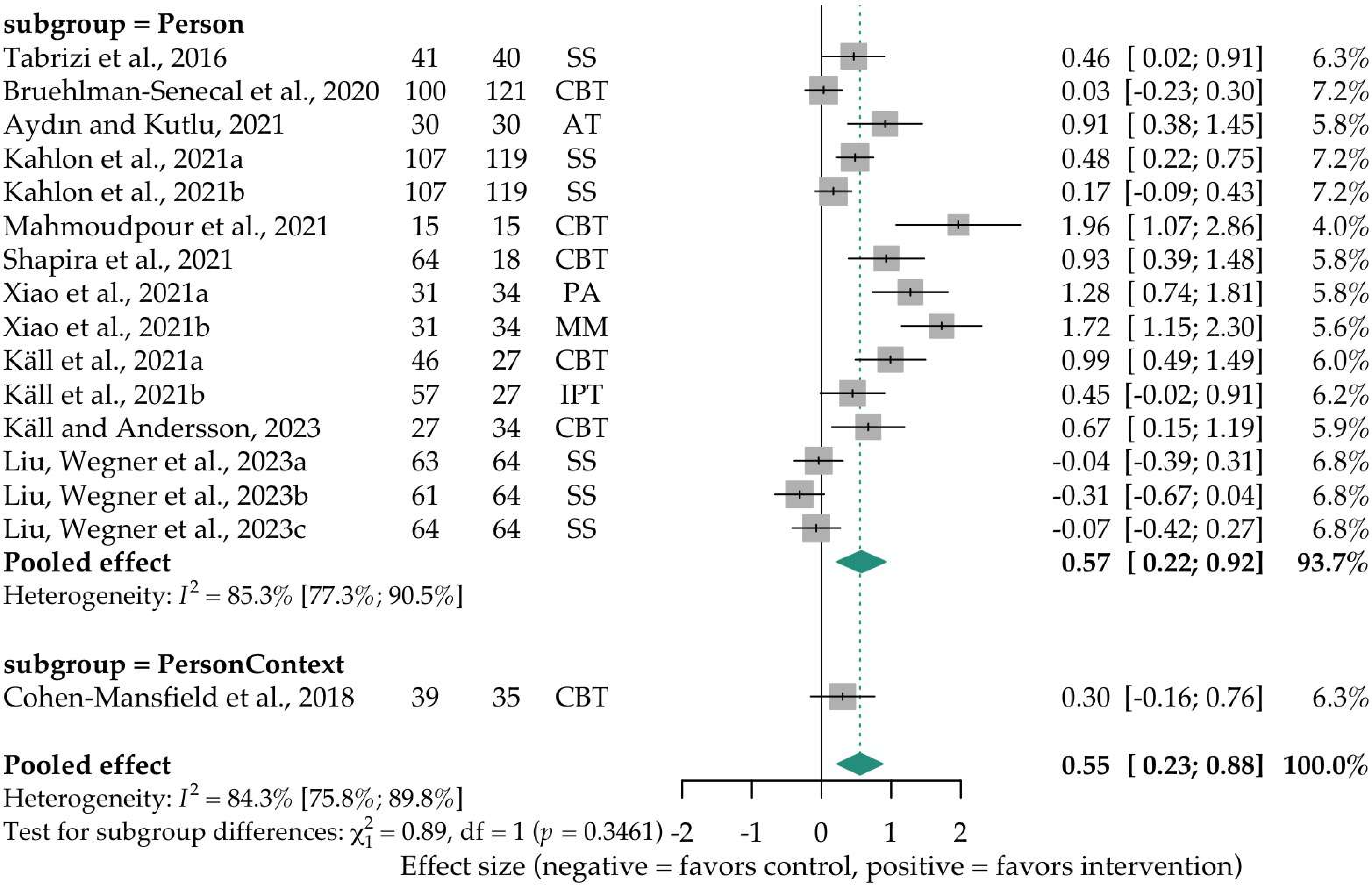

Note. Random-effects model with Hartung–Knapp adjustment for a more accurate standard error.
AC = Main Active Component used in each intervention; AT = Art Therapy; CBT = Cognitive Behavioral Therapy; CI = Confidence Interval; df = degrees of freedom; g Post = Hedges' g at post intervention; I2 = heterogeneity; IPT = Internet-based Interpersonal Therapy; MM = Mindful Movement; nExp = Experimental group sample; nCon = Control group sample; PA = Physical Activity; SS = Social Support

Figure S13

Forest plot of the effect sizes of the interventions vs. controls at post intervention (k = 16): Delivery mode subgroup analysis

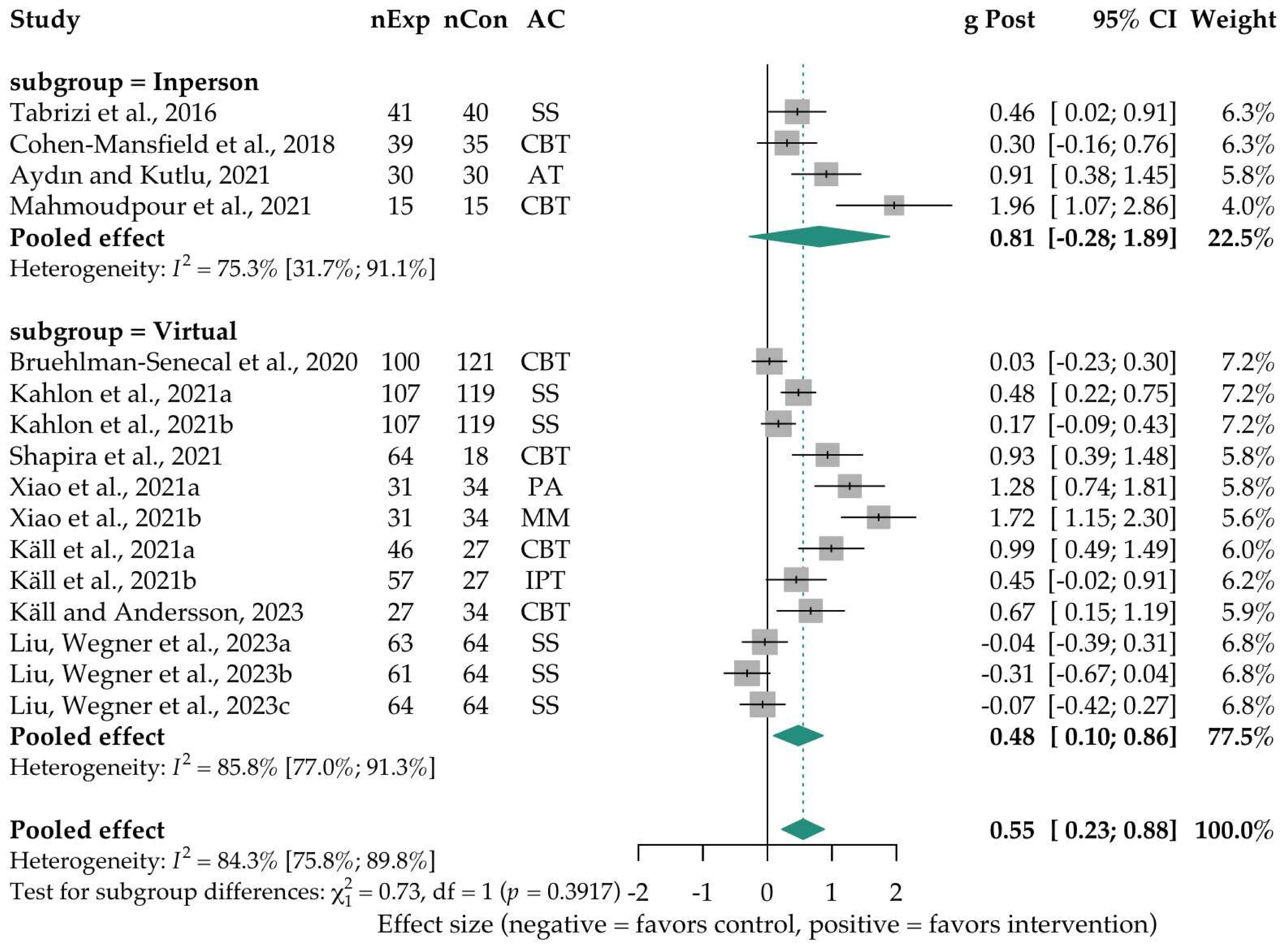

Note. Random-effects model with Hartung–Knapp adjustment for a more accurate standard error.
AC = Main Active Component used in each intervention; AT = Art Therapy; CBT = Cognitive Behavioral Therapy; CI = Confidence Interval; df = degrees of freedom; g Post = Hedges' g at post intervention; I2 = heterogeneity; IPT = Internet-based Interpersonal Therapy; MM = Mindful Movement; nExp = Experimental group sample; nCon = Control group sample; PA = Physical Activity; SS = Social Support

Figure S14

Forest plot of the effect sizes of the interventions vs. controls at post intervention (k = 16): Intervention format subgroup analysis

Study	nExp	nCon	AC	g Post	95% CI	Weight
**subgroup = Group**						
Tabrizi et al., 2016	41	40	SS	0.46	[0.02; 0.91]	6.3%
Cohen-Mansfield et al., 2018	39	35	CBT	0.30	[-0.16; 0.76]	6.3%
Aydın and Kutlu, 2021	30	30	AT	0.91	[0.38; 1.45]	5.8%
Mahmoudpour et al., 2021	15	15	CBT	1.96	[1.07; 2.86]	4.0%
Shapira et al., 2021	64	18	CBT	0.93	[0.39; 1.48]	5.8%
**Pooled effect**				**0.82**	**[0.10; 1.54]**	**28.3%**
Heterogeneity: $I^2 = 69.2\%$ [21.1%; 88.0%]						
**subgroup = Individual**						
Bruehlman-Senecal et al., 2020	100	121	CBT	0.03	[-0.23; 0.30]	7.2%
Kahlon et al., 2021a	107	119	SS	0.48	[0.22; 0.75]	7.2%
Kahlon et al., 2021b	107	119	SS	0.17	[-0.09; 0.43]	7.2%
Xiao et al., 2021a	31	34	PA	1.28	[0.74; 1.81]	5.8%
Xiao et al., 2021b	31	34	MM	1.72	[1.15; 2.30]	5.6%
Käll et al., 2021a	46	27	CBT	0.99	[0.49; 1.49]	6.0%
Käll et al., 2021b	57	27	IPT	0.45	[-0.02; 0.91]	6.2%
Käll and Andersson, 2023	27	34	CBT	0.67	[0.15; 1.19]	5.9%
Liu, Wegner et al., 2023a	63	64	SS	-0.04	[-0.39; 0.31]	6.8%
Liu, Wegner et al., 2023b	61	64	SS	-0.31	[-0.67; 0.04]	6.8%
Liu, Wegner et al., 2023c	64	64	SS	-0.07	[-0.42; 0.27]	6.8%
**Pooled effect**				**0.44**	**[0.03; 0.85]**	**71.7%**
Heterogeneity: $I^2 = 86.2\%$ [77.1%; 91.7%]						
**Pooled effect**				**0.55**	**[0.23; 0.88]**	**100.0%**
Heterogeneity: $I^2 = 84.3\%$ [75.8%; 89.8%]						
Test for subgroup differences: $\chi^2_1 = 1.39$, df = 1 ($p = 0.2387$)						

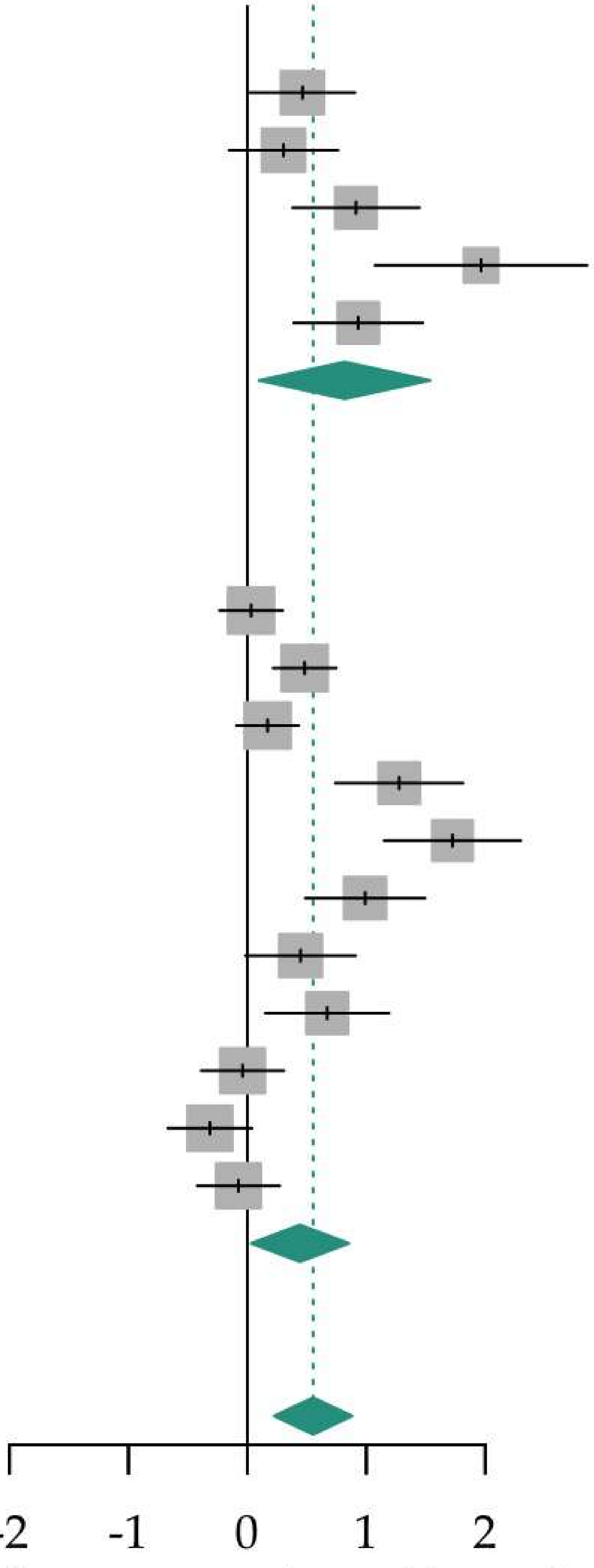

Effect size (negative = favors control, positive = favors intervention)

Note. Random-effects model with Hartung–Knapp adjustment for a more accurate standard error.
AC = Main Active Component used in each intervention; AT = Art Therapy; CBT = Cognitive Behavioral Therapy; CI = Confidence Interval; df = degrees of freedom; g Post = Hedges' g at post intervention; I2 = heterogeneity; IPT = Internet-based Interpersonal Therapy; MM = Mindful Movement; nExp = Experimental group sample; nCon = Control group sample; PA = Physical Activity; SS = Social Support

Figure S15

Forest plot of the effect sizes of the interventions vs. controls at post intervention (k = 16): Loneliness as the primary outcome subgroup analysis

Study	nExp	nCon	AC	g Post	95% CI	Weight
**subgroup = No**						
Xiao et al., 2021a	31	34	PA	1.28	[0.74; 1.81]	5.8%
Xiao et al., 2021b	31	34	MM	1.72	[1.15; 2.30]	5.6%
**Pooled effect**				**1.49**	**[-1.35; 4.33]**	**11.4%**
Heterogeneity: $I^2 = 19.7\%$						
**subgroup = Yes**						
Tabrizi et al., 2016	41	40	SS	0.46	[0.02; 0.91]	6.3%
Cohen-Mansfield et al., 2018	39	35	CBT	0.30	[-0.16; 0.76]	6.3%
Bruehlman-Senecal et al., 2020	100	121	CBT	0.03	[-0.23; 0.30]	7.2%
Aydın and Kutlu, 2021	30	30	AT	0.91	[0.38; 1.45]	5.8%
Kahlon et al., 2021a	107	119	SS	0.48	[0.22; 0.75]	7.2%
Kahlon et al., 2021b	107	119	SS	0.17	[-0.09; 0.43]	7.2%
Mahmoudpour et al., 2021	15	15	CBT	1.96	[1.07; 2.86]	4.0%
Shapira et al., 2021	64	18	CBT	0.93	[0.39; 1.48]	5.8%
Käll et al., 2021a	46	27	CBT	0.99	[0.49; 1.49]	6.0%
Käll et al., 2021b	57	27	IPT	0.45	[-0.02; 0.91]	6.2%
Käll and Andersson, 2023	27	34	CBT	0.67	[0.15; 1.19]	5.9%
Liu, Wegner et al., 2023a	63	64	SS	-0.04	[-0.39; 0.31]	6.8%
Liu, Wegner et al., 2023b	61	64	SS	-0.31	[-0.67; 0.04]	6.8%
Liu, Wegner et al., 2023c	64	64	SS	-0.07	[-0.42; 0.27]	6.8%
**Pooled effect**				**0.41**	**[0.11; 0.71]**	**88.6%**
Heterogeneity: $I^2 = 78.4\%$ [64.2%; 86.9%]						
**Pooled effect**				**0.55**	**[0.23; 0.88]**	**100.0%**
Heterogeneity: $I^2 = 84.3\%$ [75.8%; 89.8%]						
Test for subgroup differences: $\chi^2_1 = 16.80$, df = 1 ($p < 0.0001$)						

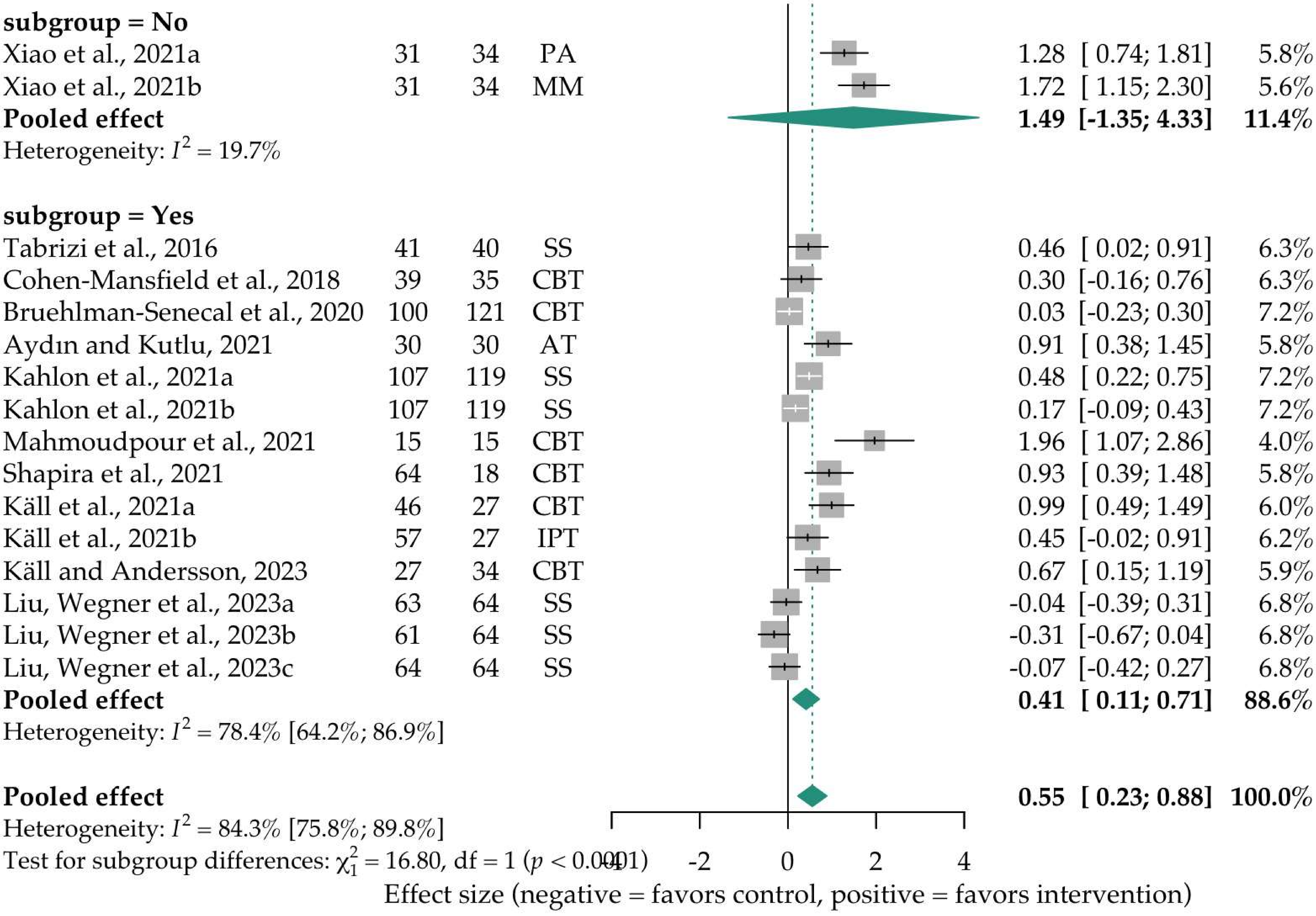

Note. Random-effects model with Hartung–Knapp adjustment for a more accurate standard error.
AC = Main Active Component used in each intervention; AT = Art Therapy; CBT = Cognitive Behavioral Therapy; CI = Confidence Interval; df = degrees of freedom; g Post = Hedges' g at post intervention; I2 = heterogeneity; IPT = Internet-based Interpersonal Therapy; MM = Mindful Movement; nExp = Experimental group sample; nCon = Control group sample; PA = Physical Activity; SS = Social Support
