# Supplementary figures and images for: Loneliness as a Public Health Challenge: A Systematic Review and Meta-Analysis to Inform Policy and Practice
Source: Eur J Investig Health Psychol Educ. 2025 Jul 11;15(7):131. doi: 10.3390/ejihpe15070131 (PMC12293955; doi:10.3390/ejihpe15070131)

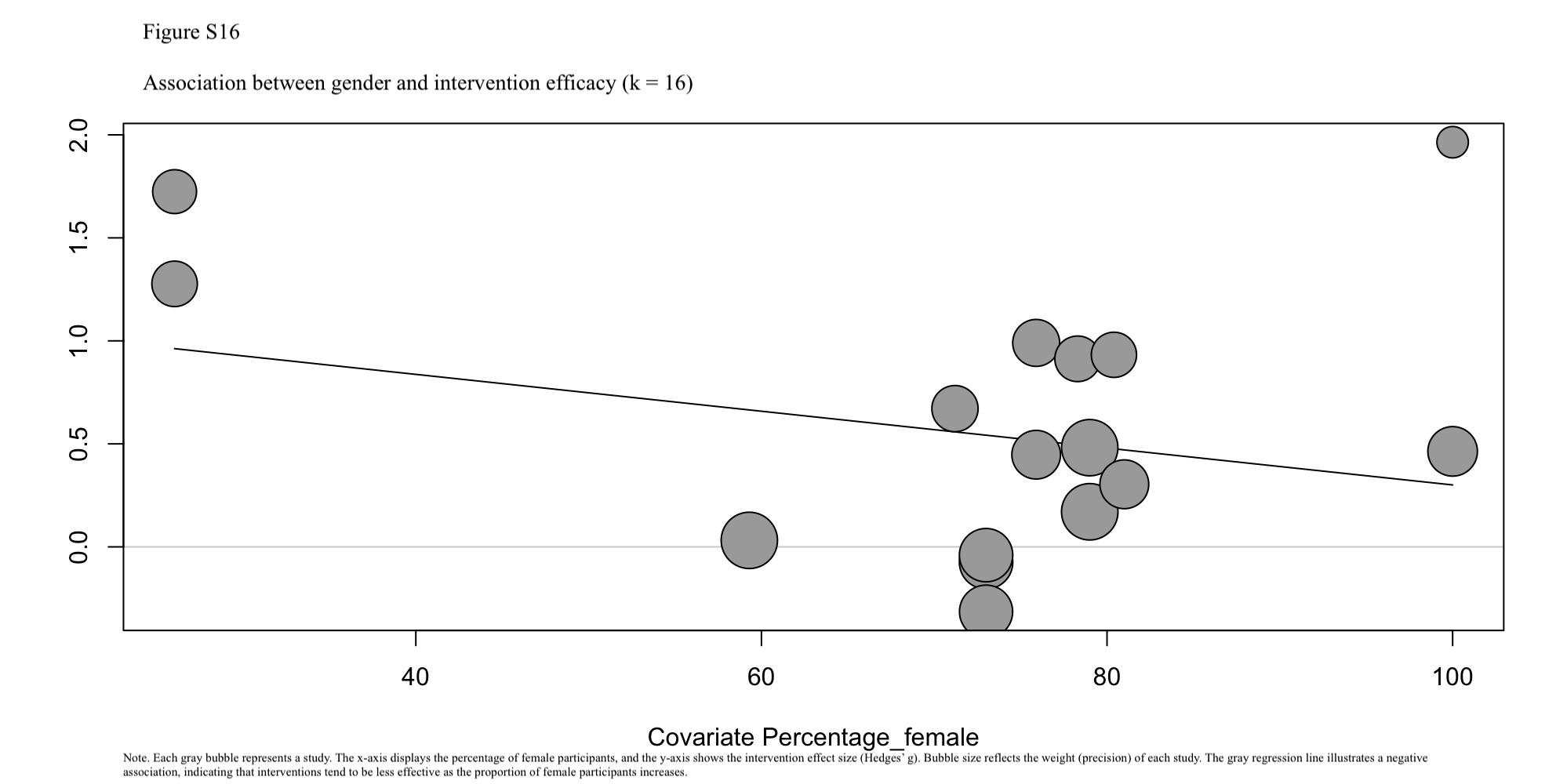

Supplement: Supplementary file 1 [file ejihpe-15-00131-s001.zip › Supplement 7_Association between gender and intervention efficacy, Figure S16.png]
